# Role of metallothionein 3 in diabetic nephropathy via interplay with HIF-1α

**DOI:** 10.1007/s13340-025-00840-y

**Published:** 2025-08-19

**Authors:** Yuri Takiyama, Yumi Takiyama, Takao Takiyama, Ryoichi Bessho, Hiroya Kitsunai, Akira Takasawa, Hiroshi Nomoto

**Affiliations:** 1 Division of Endocrinology, Metabolism and Rheumatology, Department of Medicine, Asahikawa Medical University, 2-1-1-1 Midorigaoka Higashi, Asahikawa, 078-8510 Japan; 2 Division of Tumor Pathology, Department of Pathology, Asahikawa Medical University, 2-1-1-1 Midorigaoka Higashi, Asahikawa, 078-8510 Japan

**Keywords:** Diabetic nephropathy, Metallothionein 3, HIF-1, Hypoxia

## Abstract

Metallothioneins (MTs) are a cysteine-rich protein that scavenges reactive oxygen species. Hypoxia is involved in the progression of diabetic nephropathy (DN) and aggravates oxidative stress. Hypoxia dramatically induced *MT3*, whereas induced around twofold increment in *MT2*, but inhibited *MT1* in human renal proximal tubular epithelial cells (HRPTECs). Given that the role of MT3 in DN remains unclear, we explored the involvement of MT3 in DN. Microarray analysis also identified MT3-regulated candidate genes, including ceruloplasmin (*CP*) and cytochrome b reductase 1 (*CYBRD1*), as well as FGF-Klotho (KL)-FGFR complexes in HRPTECs. Hypoxia significantly induced *MT3* expression through HIF-1-dependent mechanisms, and MT3 small interfering RNA (siRNA) decreased *CP*, *CYBRD1*, and *KL* expression under hypoxic conditions. In humanized MT3-BACTg mice, except HIF-1α, diabetes significantly increased the expression of MT3, CP, CYRBD1, FGFR2, and KL in the renal cortex in MT3-BACTg mice. Diabetic MT3-BACTg mice presented more severely damaged mitochondria in proximal tubules than their wild-type littermates did, accompanied with peritubular capillary obstruction by swollen endothelial cells. Moreover, the proximal tubules-specific overexpression of MT3 in mice (MT3Tg) represented no overlap in the protein expression between MT3 and HIF-1α in diabetic kidney. Accordingly*,* MT3 siRNA significantly augmented HIF-1α protein and *HIF1A* in HRPTECs. Finally, *MT3* expression in the renal tubulointerstitium was positively correlated with the glomerular filtration rate (GFR) in DN subjects by data from Nephroseq. In conclusion, these results showed that there might be a unique interplay between MT3 and HIF-1α in diabetic kidney of to regulate hypoxia-induced HIF-1α expression.

**Supplementary Information:**

The online version contains supplementary material available at 10.1007/s13340-025-00840-y.

## Introduction

Diabetic nephropathy (DN) is known to be a leading cause of end-stage renal failure worldwide. Chronic hypoxia is one of the underlying mechanisms involved in the pathogenesis of DN [1], especially relative hypoxia in early stage of DN [2]. Renal hypoxia increases oxidative stress; in turn, oxidative stress conversely exacerbates renal hypoxia [3]. Oxidative stress is caused by an imbalance between the production of reactive oxygen species (ROS) and the function of antioxidant proteins [3]. Several antioxidant proteins, such as nuclear factor erythroid 2-related factor 2 (Nrf2, gene name NFE2 like bZIP transcription factor 2; *NFE2L2*) [4, 5], uncoupling proteins [6], and manganese superoxide dismutase [7], have been shown to prevent DN by inhibiting oxidative stress.

Metallothionein 3 (MT3) is a cysteine-rich protein and an antioxidant with a low molecular weight (6.927 kDa). MT3 scavenges ROS and protects cells from oxidative stress by binding redox-active metals, such as Cu(I) and Zn(II) [8]. MT3 is one isoform of the four MTs, three isoforms except MT4 have been identified in the mammalian kidneys [9]. MT3 was originally identified as a neuronal growth inhibitory factor that is deficient in the brain in Alzheimer’s disease [10]. Previous studies demonstrated that MT3 is also expressed in the human kidney [11, 12]. Of note, MT3 mRNA expression is much greater than that of the housekeeping gene *β*-actin in especially the proximal tubules [13]. In addition, MT3 specifically induces the dome formation in immortalized human proximal tubular cells (HK-2 cell line) and is involved in the transport function [13]. MT3 interacts with proteins involved in cytoskeletal organization (*β*-actin, myosin, and tropomyosin-3) and energy metabolism (glycolytic enzymes; enolase 1 and aldolase A) [14]. The C-terminal sequence of *MT3*, which is not present of any other member of the MT gene, is involved in a reversal of the epithelial-mesenchymal transition process (MET) in HK-2 cells [15]. Intriguingly, a very recent study showed that MT3 is the most abundantly expressed at both the transcript and protein levels in response to insulin resistance specifically in proximal tubular cells [16]. Notwithstanding these previous findings regarding MT3, its role in DN is not known.

In this study, we determined the involvement of MT3 in the pathogenesis of DN using human renal proximal tubular epithelial cells (HRPTECs) and transgenic mice harboring a bacterial artificial chromosome (BAC) expressing human MT3 mRNA and protein to generate humanized BAC transgenic mice (MT3-BACTg) and proximal tubule-specific overexpressed human *MT3* transgenic mice (MT3Tg).

## Methods

### Animal models

All animal experiments conformed to the National Health Guide for the Care and Use of Laboratory Animals and were approved by the Research Center for Animal Life Science at Asahikawa Medical University (Approval No. 23–03, Date of Approval; March 14, 2023). Bacterial artificial chromosome transgenic (BACTg) model mice expressing human MT3 mice (MT3-BACTg) and proximal tubule-specific human *MT3* transgenic mice (MT3Tg) were generated via a BAC clone (ID: RP11-933M22) harboring the human MT3 gene and the mouse sglt2 promoter for targeting human *MT3* gene to renal proximal tubular cells by Unitech Co. (Chiba, Japan), respectively. Wild-type littermates were used as controls in all the experiments. All the mice were housed with free access to food and water on a 12/12 h light/dark cycle. The male MT3-BACTg mice and MT3Tg mice were divided into two groups: a group that received no treatment and a group that received streptozotocin (STZ; 55 mg/kg, i.p.) for 5 days at 6 weeks of age. At 15 weeks of age, we evaluated the metabolic parameters, renal function and pathological findings of the kidneys.

### Statistical analysis

The sample sizes for the animal studies were determined according to a previous publication [17]. At least three separate experiments were performed per protocol. Each treatment group was assayed in duplicate for real-time RT-PCR. Values are presented as the means ± standard deviations (SDs). Measured variables for mouse urinary albumin and NGAL were log(e) transformed. The significance of the differences between groups was determined via unpaired Student’s t tests and one-way repeated-measures analysis of variance (ANOVA) with Bonferroni post hoc correction for multiple comparisons as needed. For nonparametric tests, statistical analysis was performed via the Mann‒Whitney *U* test. Values of *p* < 0.05 were considered statistically significant. All analyses were performed via GraphPad Prism ver. 7.0 software (Boston, MA, USA; https://www.graphpad.com). Other detailed experimental methods are included in the Supplementary Methods.

## Results

### Hypoxia induced *MT2* and *MT3* in HRPTECs

**Fig. 1:**
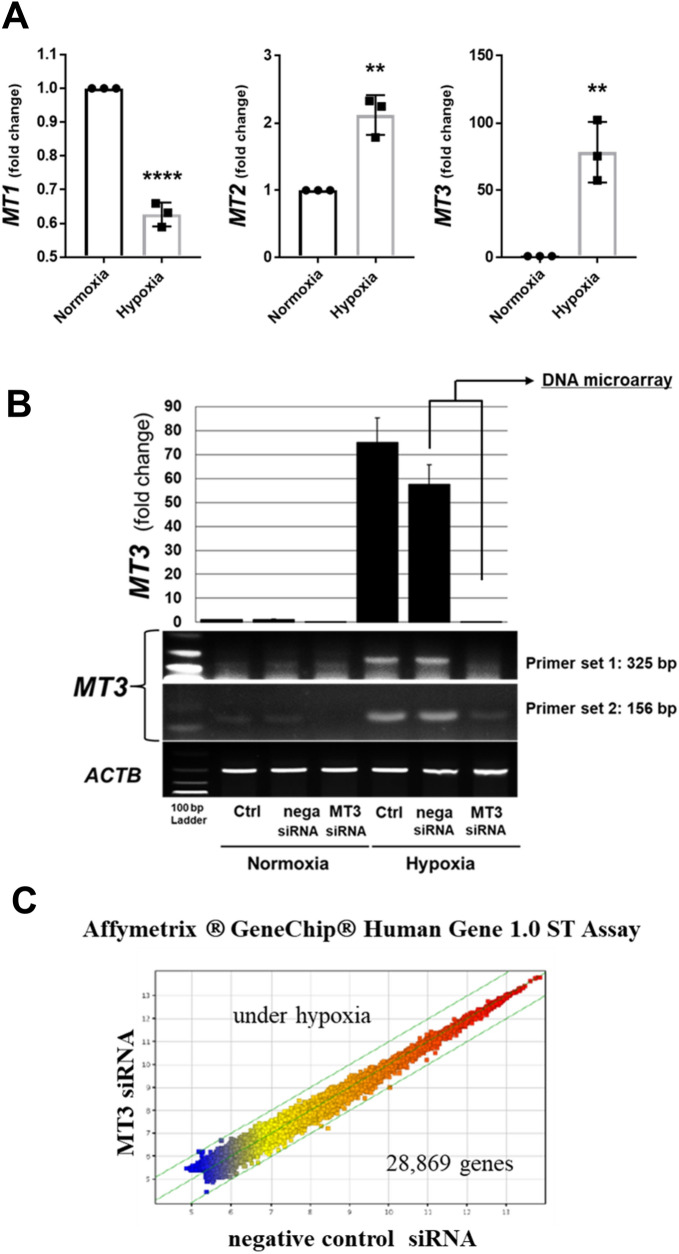
Identification of MT3 as hypoxia-induced gene dependent on HIF-1α. **A** qRT-PCR showed that hypoxia induced *MT2* and *MT3*, not *MT1,* in HRPTECs. **p < 0.01, ****p < 0.0001 *vs*. the normoxia control. **B** HRPTECs were transiently transfected with a negative control, MT3-specific siRNAs (25 nmol/L final level). Twenty-four hours after transfection, the cells were cultured under normoxic (21% O_2_) and hypoxic (1% O_2_) conditions overnight. Real-time reverse transcriptase PCR (RT-PCR) (upper panel) and conventional RT-PCR (lower panel) with two primer sets (Supplementary Table 1) revealed that MT3 siRNA efficiently decreased *MT3* expression in HRPTECs. **C** Gene expression microarray analysis of MT3-knockdown HRPTECs under hypoxia. To determine which molecules are regulated by MT3 in human renal proximal tubular cells, we analyzed microarray data via an Affymetrix GeneChip (Human Gene 1.0 ST Array) with siRNA-mediated knockdown of MT3 under hypoxia in HRPTECs

We evaluated the effects of hypoxia on the expressions of MT1, 2 and 3 in HRPTECs. Hypoxia significantly decreased *MT1* (0.63-fold *vs.* normoxia control, *p* < 0.0001) and increased *MT2* (2.12-fold,* p* < 0.01) and *MT3* (78.28-fold, *p* < 0.01) levels in HRPTECs (Fig. 1A). Given the high hypoxia-induced increment in *MT3* expression in HRPTECs, we focused on the role of MT3 in hypoxia-involved DN in the current study.

### MT3 siRNA decreases the expression of DN-related genes in HRPTECs

We confirmed that hypoxia significantly induced *MT3* expression in HRPTECs, as evaluated via qRT-PCR and conventional RT-PCR (Fig. 1B). We compared gene expression between the control and MT3-knockdown HRPTECs under hypoxic conditions via Affymetrix Human Gene 1.0 ST arrays (Fig. 1C). DNA microarray analysis revealed a − 1.5638772-fold change in *MT3* expression in *MT3* siRNA-treated HRPTECs compared with that in negative control siRNA-treated HRPTECs; thus, the threshold for linear fold change absolute values was set to > 1.5 to cover both sides, as shown in Table 1. Among the downregulated genes, we focused on the top 3 downregulated genes: ceruloplasmin (*CP*), cytochrome b reductase 1 (*CYBRD1*), and fibroblast growth factor receptor 2 (*FGFR2*).

**Table 1:** The changed over 1.5-fold by MT3 siRNA under hypoxic condition in HRPTECs

Ref seq	Gene symbol	Gene description	Fold change	Regulation
NM_001136233|NM_001136234	FAM48B2|FAM48B1	Family with sequence similarity 48, member B2 | family with sequence similarity 48, member B1	1.9664215	up
NM_139174|NM_001145400	ADAD2|SFPQ	Adenosine deaminase domain containing 2 | splicing factor proline and glutamine rich	1.701523	up
NR_026800	KIAA0125|FAM30A	KIAA0125 | family with sequence similarity 30, member A	1.6931752	up
NM_005371|NM_023033	METTL1	Methyltransferase like 1	1.6662333	up
NM_001001394	DNAJB3	DnaJ (Hsp40) homolog, subfamily B, member 3	1.5994776	up
NM_001004466	OR10H5	olfactory receptor, family 10, subfamily H, member 5	1.5759735	up
NM_001097	ACR	Acrosin	1.560273	up
NM_172239	REXO1L1	REX1, RNA exonuclease 1 homolog (S. cerevisiae)-like 1	1.5201751	up
NR_029478|NR_027033	MIRLET7A3	MicroRNA let-7a-3	1.5128531	up
NM_020678	LRTM1	Leucine-rich repeats and transmembrane domains 1	1.5127611	up
NM_005390	PDHA2	Pyruvate dehydrogenase (lipoamide) alpha 2	1.5106342	up
NM_033050	SUCNR1	succinate receptor 1	1.5078278	up
NM_138296	PTCRA	Pre T-cell antigen receptor alpha	1.5042478	up
NM_198993	STAC2	SH3 and cysteine-rich domain 2	1.5017763	up
NM_000096	CP	Ceruloplasmin (ferroxidase)	− 2.3331416	down
NM_024843|NM_001127383	CYBRD1	Cytochrome b reductase 1	− 2.0188644	down
NM_000141|NM_022970|NM_001144915|NM_001144916|NM_001144918|NM_001144913|NM_001144917|NM_001144914|NM_001144919	FGFR2	Fibroblast growth factor receptor 2	− 1.8946843	down
NM_001075|NM_001144767	UGT2B10	UDP glucuronosyltransferase 2 family, polypeptide B10	− 1.839555	down
NM_003480	MFAP5	Microfibrillar associated protein 5	− 1.8390621	down
NM_015429	ABI3BP	ABI family, member 3 (NESH) binding protein	− 1.7669579	down
NM_001003702	ARHGEF35	Rho guanine nucleotide exchange factor (GEF) 35	− 1.7565662	down
NM_001137550	LRRFIP1	Leucine-rich repeat (in FLII) interacting protein 1	− 1.7175369	down
NM_003641	IFITM1	Interferon induced transmembrane protein 1 (9–27)	− 1.7062886	down
NR_002746	SNORD47|GAS5	Small nucleolar RNA, C/D box 47 | growth arrest-specific 5 (non-protein coding)	− 1.6858332	down
NM_017938|NM_001104544|NM_001104545	FAM70A	Family with sequence similarity 70, member A	− 1.6848774	down
NR_002562	SNORD28	Small nucleolar RNA, C/D box 28	− 1.6834495	down
NM_031940|NM_001024380|NM_001024381|NM_078473	TM2D2	TM2 domain containing 2	− 1.680962	down
NM_002038|NM_022872|NM_022873	IFI6	Interferon, alpha-inducible protein 6	− 1.6772653	down
NM_033655	CNTNAP3|CNTNAP3B	Contactin-associated protein-like 3 | contactin-associated protein-like 3B	− 1.6638206	down
NM_012086	GTF3C3	General transcription factor IIIC, polypeptide 3, 102 kDa	− 1.6591194	down
NM_003064	SLPI	Secretory leukocyte peptidase inhibitor	− 1.6463447	down
NM_033655	CNTNAP3|CNTNAP3B	Contactin-associated protein-like 3 | contactin-associated protein-like 3B	− 1.6300697	down
NM_001014380|NM_032116	KATNAL1	Katanin p60 subunit A-like 1	− 1.625215	down
NM_006174	NPY5R	Neuropeptide Y receptor Y5	− 1.6185701	down
NM_015975	TAF9B	TATA-box-binding protein associated factor 9b	− 1.6144912	down
NM_144777|NM_003843|NM_ 001160706	SCEL	Sciellin	− 1.6074374	down
NM_018365	MNS1	Meiosis-specific nuclear structural 1	− 1.5979369	down
NM_001677|NM_013330	ATP1B1|NME7	ATPase, Na +/K + transporting, beta 1 polypeptide | non-metastatic cells 7, protein expressed in (nucleoside-diphosphate kinase)	− 1.5899302	down
NM_002016	FLG	Filaggrin	− 1.5722692	down
NR_002750	SNORD44	Small nucleolar RNA, C/D box 44	− 1.5653511	down
NM_005954	MT3	**Metallothionein 3**	− 1.5638772	down
NR_015379	UCA1	Urothelial cancer associated 1 (non-protein coding)	− 1.5607672	down
NM_004598	SPOCK1	Sparc/osteonectin, cwcv and kazal-like domains proteoglycan (testican) 1	− 1.5567142	down
NM_145016	GLYATL2	Glycine-N-acyltransferase-like 2	− 1.5551503	down
NM_000489|NM_138270	ATRX	Alpha thalassemia/mental retardation syndrome X-linked (RAD54 homolog, S. cerevisiae)	− 1.5537893	down
NM_206996	SPAG17	Sperm associated antigen 17	− 1.5520457	down
NM_004666	VNN1	Vanin 1	− 1.5513672	down
NM_201442|NM_001734	C1S	Complement component 1, s subcomponent	− 1.5486898	down
NM_025257|NM_001178044|NM _001178045|NM_000434	SLC44A4|NEU1	Solute carrier family 44, member 4 | sialidase 1 (lysosomal sialidase)	− 1.544443	down
NM_018369|NM_001145208	DEPDC1B	DEP domain containing 1B	− 1.5434974	down
NM_004362|NM_001130675	CLGN	calmegin	− 1.543249	down
NM_025257|NM_001178044|NM _001178045|NM_000434	SLC44A4|NEU1	Solute carrier family 44, member 4 | sialidase 1 (lysosomal sialidase)	− 1.541748	down
NM_013390|NM_001135820	TMEM2	Transmembrane protein 2	− 1.5411102	down
NM_030572	C12orf39	Chromosome 12 open reading frame 39	− 1.5407965	down
NR_033438|NM_020353|NR_03 3439|NM_001128306|NM_00117 7304	PLSCR4	Phospholipid scramblase 4	− 1.5393462	down
NM_000860|NM_001145816|NR _027332	HPGD	Hydroxyprostaglandin dehydrogenase 15-(NAD)	− 1.5356336	down
NM_004365	CETN3	Centrin, EF-hand protein, 3 (CDC31 homolog, yeast)	− 1.5286751	down
NM_033171|NM_033172|NM_03 3173|NM_006057|NM_033170	B3GALT5	UDP-Gal:betaGlcNAc beta 1,3-galactosyltransferase, polypeptide 5	− 1.5253178	down
NM_024685	BBS10	Bardet-Biedl syndrome 10	− 1.5236772	down
NM_006180|NM_001018064| NM_001018065|NM_001007 097|NM _001018066	NTRK2	Neurotrophic tyrosine kinase, receptor, type 2	− 1.521899	down
NM_145343|NM_003661|NM _001136540|NM_001136541	APOL1	Apolipoprotein L, 1	− 1.5189677	down
NM_002153	HSD17B2	Hydroxysteroid (17-beta) dehydrogenase 2	− 1.5185025	down
NM_020234|NM_001144955	DTWD1	DTW domain containing 1	− 1.516844	down
NM_001920|NM_133503|NM _133504|NM_133505|NM_13 3506|NM_133507	DCN	Decorin	− 1.5146513	down
NR_002737	SNORD59A	Small nucleolar RNA, C/D box 59A	− 1.5123222	down
NM_001010915|NM_017794	PTPLAD2|KIAA1797	Protein tyrosine phosphatase-like A domain containing 2 | KIAA1797	− 1.5115247	down
NM_017996|NM_001144074| NR_026645	DET1	De-etiolated homolog 1 (Arabidopsis)	− 1.5097446	down
NR_033192|NM_014167	CCDC59	Coiled-coil domain containing 59	− 1.5092642	down
NM_018487	TMEM176A	Transmembrane protein 176A	− 1.5091854	down
NR_002748	SNORD45B|RABGGTB	Small nucleolar RNA, C/D box 45B | Rab geranylgeranyltransferase, beta subunit	− 1.5069065	down
NM_016338|NM_001134779	IPO11	Importin 11	− 1.5067282	down
NM_207333	ZNF320	Zinc finger protein 320	− 1.5062457	down

### HIF-1α and Nrf2 regulate *MT3* under hypoxia in HRPTECs

**Fig. 2:**
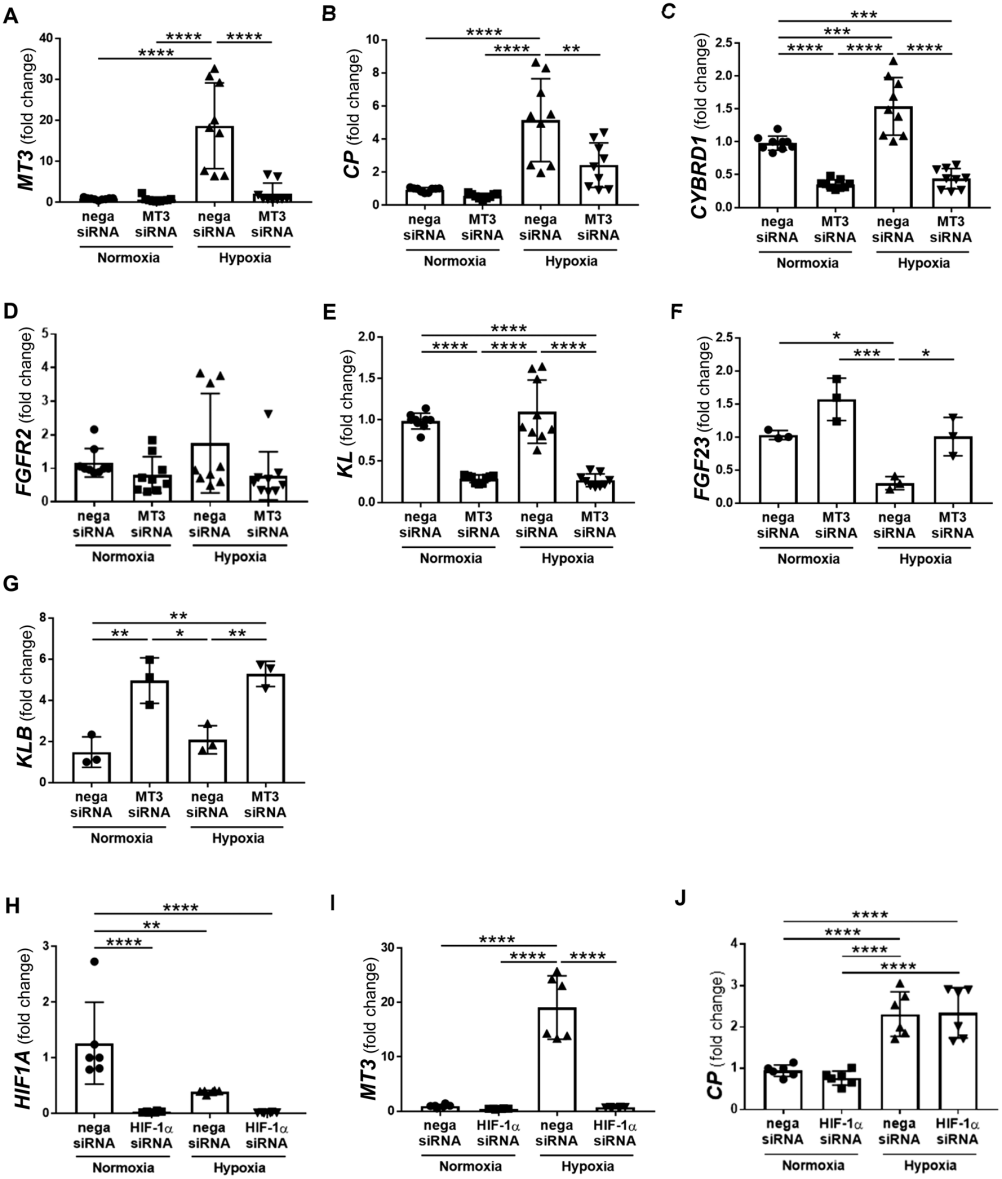

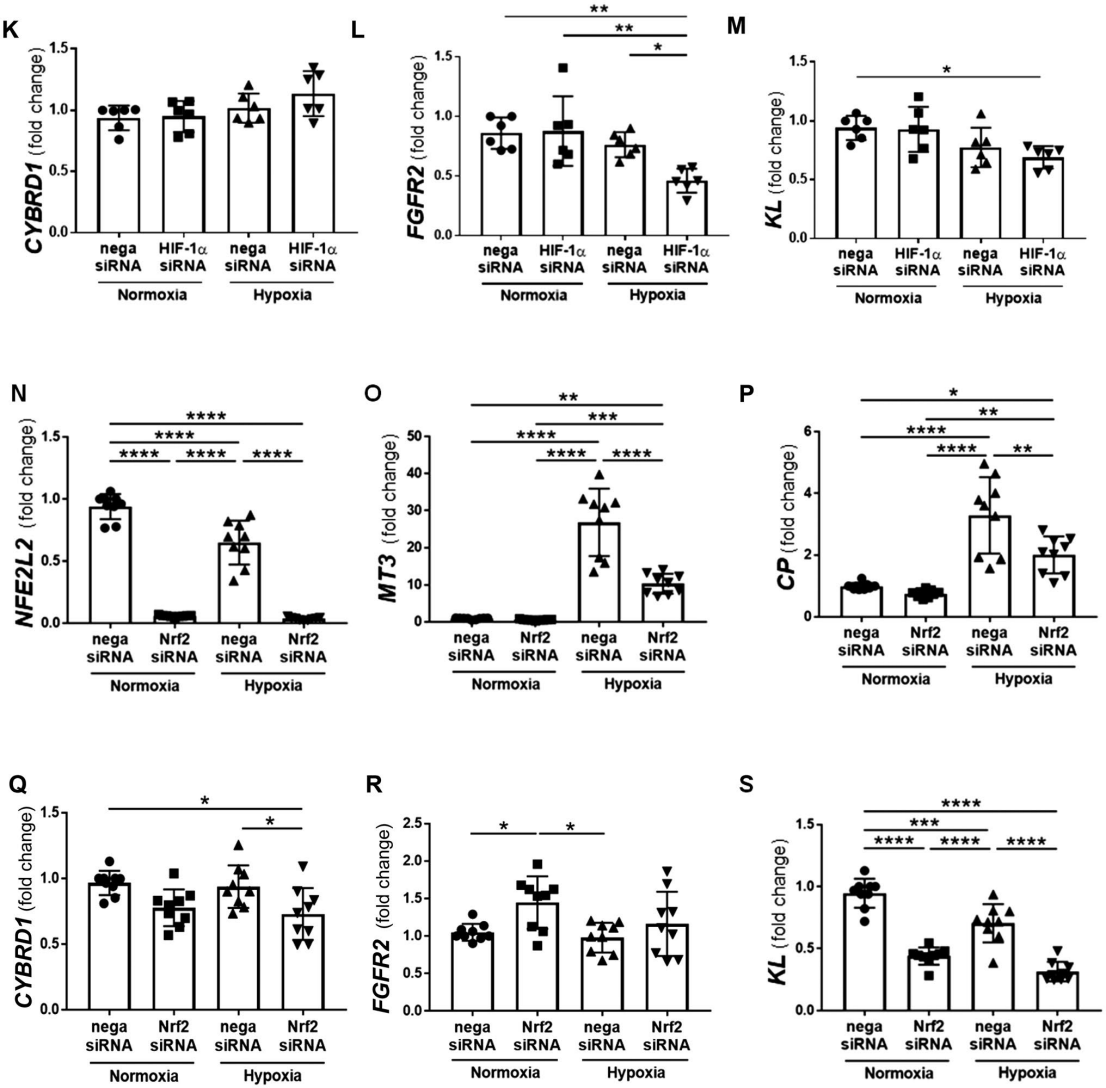
Regulation of *MT3* expression and downstream genes in human renal proximal tubular epithelial cells (HRPTECs). **A** Hypoxia significantly induced *MT3* expression, and MT3 siRNA efficiently decreased *MT3* expression in HRPTECs. MT3 siRNA significantly inhibited hypoxia-induced *CP* **B** and *CYBRD1* **C** expression. **D** Hypoxia did not significantly increase *FGFR2* expression because of wide variation. MT3 siRNA tended to decrease *FGFR2* expression independent of oxygen conditions. **E** Oxygen conditions did not affect *KL* expression, but MT3 siRNA significantly and equally decreased *KL* expression under normoxia and hypoxia. **F** In contrast to the results for the genes described above, hypoxia decreased *FGF23* expression and MT3 siRNA increased *FGF23* expression. **G** Oxygen conditions did not change *KLB* expression in the same manner as *KL* expression, but MT3 siRNA increased *KLB* expression but not *KL* expression*.* **H** Hypoxia inducible factor (HIF)−1α siRNA efficiently inhibited *HIF1A* compared with negative siRNA under normoxic condition. As hypoxia alone significantly inhibited *HIF1A* compared with those in normoxic conditions, HIF-1α siRNA tended toward decrease in *HIF1A* expression under hypoxia. **I** *HIF1A* siRNA drastically decreased hypoxia-induced *MT3* expression. Except for *FGFR2* expression **L**, *HIF1A* siRNA did not decrease *CP* **J**, *CYRBD1* **K,** or *KL* **M** expression. **N** Hypoxia significantly decreased *NFE2L2* expression. Conformingly, Nrf2 siRNA decreased *NFE2L2* expression. Nrf2 siRNA also decreased *MT3* **O**, *CP* **P**, *CYBRD1* **Q**, *FGFR2* **R,** and *KL* **S** expression. Statistical comparisons were performed via one-way analysis of variance ANOVA with post hoc Bonferroni multiple comparison tests. *p < 0.05, **p < 0.01, ***p < 0.001, and ****p < 0.0001

To confirm the results of the microarray analysis, we analyzed the regulation of *MT3* expression via qRT-PCR. MT3 mRNA expression was significantly upregulated by 21.13-fold in HRPTECs under hypoxia compared with control siRNA-treated HRPTECs under normoxia (Fig. 2A). Hypoxia induces ROS, which activate transcription factors, such as hypoxia-inducible factor 1α (HIF-1α) and Nrf2 [19]. Because HIF-1α can bind to the HRE, we screened a region of the promoter region of the human *MT3* gene and searched for transcriptional start sites for HIF via JASPAR (profile score threshold 80%, https://jaspar.elixir.no/). The JASPAR analysis revealed potential binding sites for HIF1A in the promoter of human *MT3*, indicating that human *MT3* could be a direct transcriptional target of HIF (Supplementary Table 5). We also detected fewer putative site(s) for HIF1A in the promoter region of the mouse *Mt3* gene than human *MT3* (Supplementary Table 6). Because of CTG triplet repeats in the promoter region, which function as negative elements, hypoxia failed to induce expression of the *Mt3* gene in cultured mouse proximal tubular cells (Supplementary Fig. 1).

Accordingly, compared with control siRNA, HIF-1α siRNA markedly decreased *MT3* expression under hypoxia in HRPTECs (Fig. 2I). Nrf2 is another major redox-responsive molecule that regulates genes involved in oxidative stress and inflammation. *MT3* expression was also significantly downregulated by Nrf2 siRNA under hypoxia (Fig. 2O). In addition, the antioxidant N-acetylcysteine (NAC) failed to restore hypoxia-induced *MT3* expression, whereas the mitochondrial respiratory inhibitor NADPH oxidase (NOX) abolished the stimulatory effect of hypoxia on *MT3* expression (Supplementary Fig. 2), indicating that hypoxia-induced *MT3* expression is dependent on the mitochondrial respiratory system or NOX.

### MT3 and Nrf2, but not HIF-1α, siRNAs decrease *CP*, *CYBRD1* and* KL* expression in HRPTECs

**Fig. 3:**
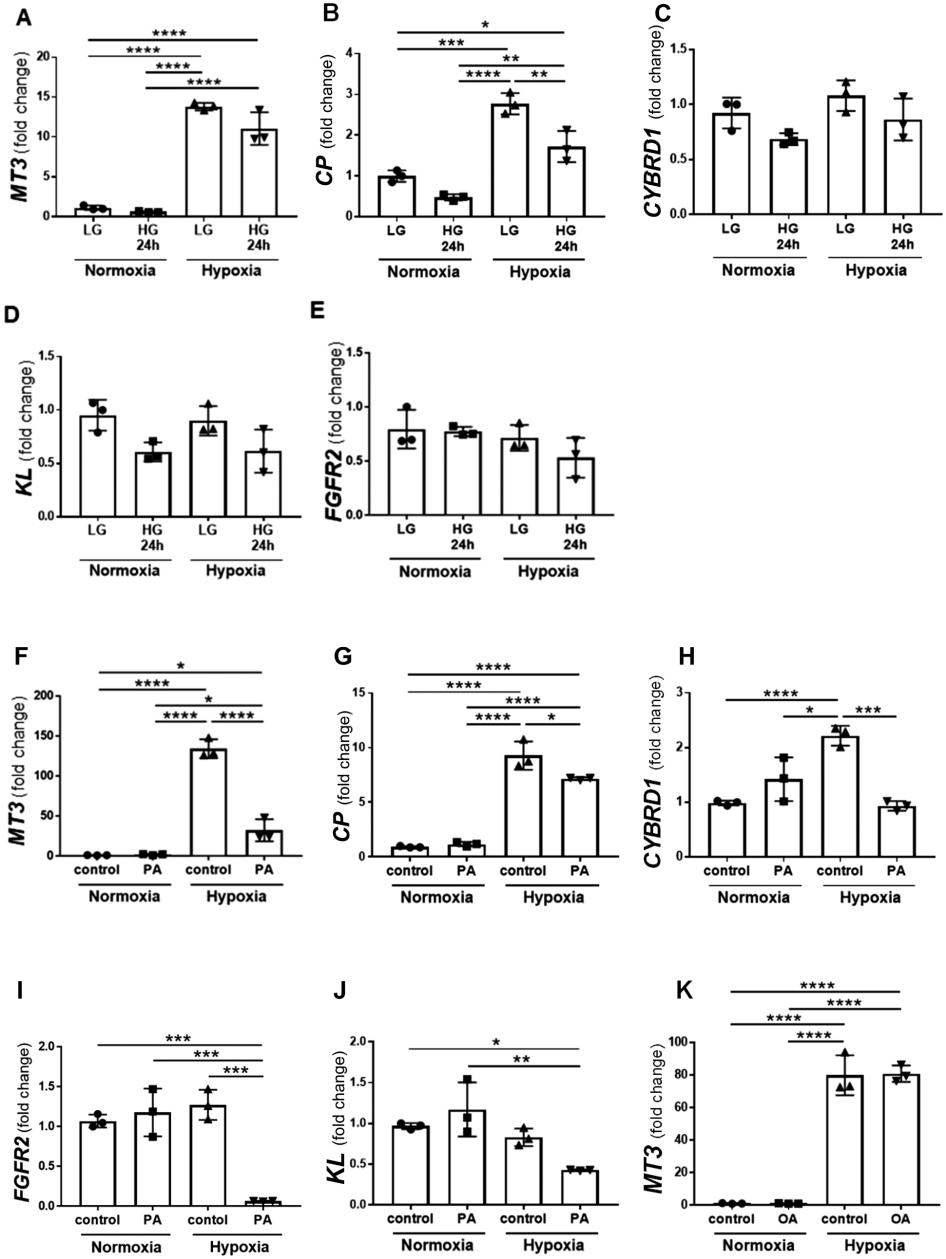
Effects of mimicking the diabetic microenvironment on the expression of MT3 and its downstream genes in human renal proximal tubular epithelial cells (HRPTECs). High glucose (D-glucose, 25.5 mM) significantly decreased hypoxia-induced *CP* expression **B** but failed to decrease *MT3* **A**, *CYBRD1* **C**, *KL* **D,** or *FGFR2* **E** expression. In contrast, palmitic acid (PA; 150 μM) decreased *MT3* **F**, *CP* **G**, *CYBRD1* **H**, *FGFR2* **I,** and *KL* **J** expression. **K** Oleic acid (OA; 150 μM) did not affect *MT3* expression under normoxia or hypoxia. Statistical comparisons were performed via one-way analysis of variance (ANOVA) with post hoc Bonferroni multiple comparison tests. *p < 0.05, **p < 0.01, ***p < 0.001 and ****p < 0.0001

Consistent with the results of the microarray analysis, MT3 siRNA significantly decreased hypoxia-induced *CP* expression (Fig. 2B). Regarding *CYBRD1* expression, hypoxia produced various effects because of its limited ability and the use of primary cultured cells derived from different human donors (Figs. 2C and 3C). Hypoxia failed to induce *FGFR2* expression, and MT3 siRNA just tended to decrease *FGFR2* expression (Fig. 2D). We also analyzed the mRNA expression of the FGF–Klotho–FGFR complex. MT3 siRNA significantly decreased *KL* expression under both normoxia and hypoxia (Fig. 2E). In contrast, MT3 siRNA significantly increased *FGF23* expression under hypoxia (Fig. 2F) and the expression of *β*-klotho (*KLB*), which shares 41.2% amino acid identity with KL, under both normoxia and hypoxia (Fig. 2G), suggesting the potential regulation of FGF‒KL‒FGFR complexes, such as mutual negative feedback.

HIF-1α siRNA significantly decreased *MT3* expression, but not *CP*, *CYBRD1* or *KL* expression (Fig. 2J, K, M), indicating that these three genes are directly regulated by MT3, not HIF-1α, under hypoxia. On the other hand, Nrf2 siRNA significantly decreased *CP* and *CYBRD1* expression under hypoxia (Fig. 2P and Q) and *KL* expression under both normoxia and hypoxia (Fig. 2S). In contrast, Nrf2 siRNA significantly increased *FGFR2* expression under normoxia (Fig. 2R), suggesting the complicated regulation in FGFR/KL complexes.

### PA, not oleic acid or high glucose, inhibits hypoxia-induced MT3 mRNA in HRPTECs

To study the effects of glucotoxicity and lipotoxicity in diabetes, we analyzed *MT3*, *CP*, *CYBRD1*, *FGFR2,* and *KL* expression under conditions of high glucose (HG; 25.5 mM) and cytotoxic saturated free fatty acid (FFA) palmitic acid (PA) (150 μM) supplementation (Supplementary methods). HG conditions did not affect the expression of *MT3*, *CYBRD1*, *FGFR2* or *KL* (Fig. 3A, C–E) but did affect the expression of *CP* under hypoxia (Fig. 3B). In contrast, PA supplementation decreased the expression of *MT3*, *CP*, *CYBRD1*, and *FGFR2* under hypoxia (Fig. 3F–I) and tended to decrease *KL* expression under hypoxia (*p* = 0.0883) (Fig. 3J). In contrast, supplementation with oleic acid, an unsaturated fatty acid, did not change *MT3* expression (Fig. 3K).

### STZ-induced diabetic MT3-BACTg mice present increased plasma and urinary zinc levels

**Fig. 4:**
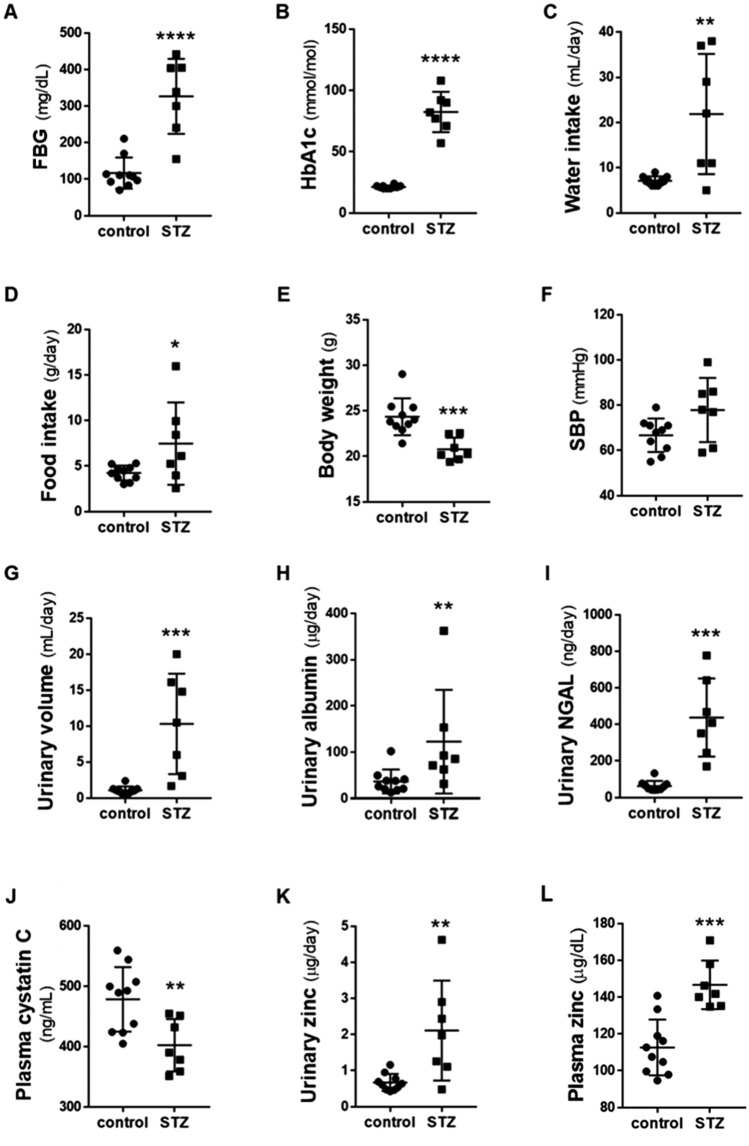
Streptozotocin-induced diabetic humanized bacterial artificial chromosome transgenic mice (MT3-BACTg mice) presented increased plasma zinc levels and hyperzincuria accompanied by **diabetic** nephropathy. **A** Fasting blood glucose (FBG). **B** Glycated hemoglobin (HbA1c). **C** Water intake for 24 h. **D** Food intake for 24 h. **E** Body weight. **F** Systolic blood pressure (SBP). **G** Urinary volume for 24 h. **H** Urinary albumin per day. **I** Urinary NGAL per day. **J** Plasma cystatin C. **K** Urinary zinc per day. **L** Plasma zinc level. Black circles: control MT3-BACTg (*n* = 10). Black squares: streptozotocin-induced diabetic MT3-BACTg mice (*n* = 7). Statistical comparisons were performed via Student’s t tests or the Mann‒Whitney U test. Urinary albumin and NGAL were log(e)transformed for statistical analysis. *p < 0.05, **p < 0.01, ***p < 0.001 and ****p < 0.0001

We then investigated the effects of MT3 on DN using STZ-induced diabetic MT3-BACTg mice (Fig. 4). The STZ-induced diabetic MT3-BACTg mice presented increased fasting blood glucose levels (*p* < 0.001), HbA1c levels (*p* < 0.001), water intake (*p* < 0.05), and food intake (*p* < 0.05) but decreased body weight (*p* < 0.001). Diabetes did not change SBP. Compared with control mice, diabetic mice presented polyuria (*p* < 0.0001), increased urinary albumin excretion (*p* < 0.01), and urinary neutrophil gelatinase-associated lipocalin (NGAL) excretion (*p* < 0.001) concomitant with hyperglycemia and polydipsia. The plasma cystatin C level was decreased (*p* < 0.01) in the STZ group compared with the control group, indicating hyperglycemia-induced hyperfiltration. Additionally, urinary zinc excretion (*p* < 0.01) and the plasma zinc level (*p* < 0.001) were increased in the STZ group compared with the control group.

### MT3 expression is increased in the renal tubules of diabetic mice, accompanied by CP, CYRBD1, FGFR2, and KL protein expression

**Fig. 5:**
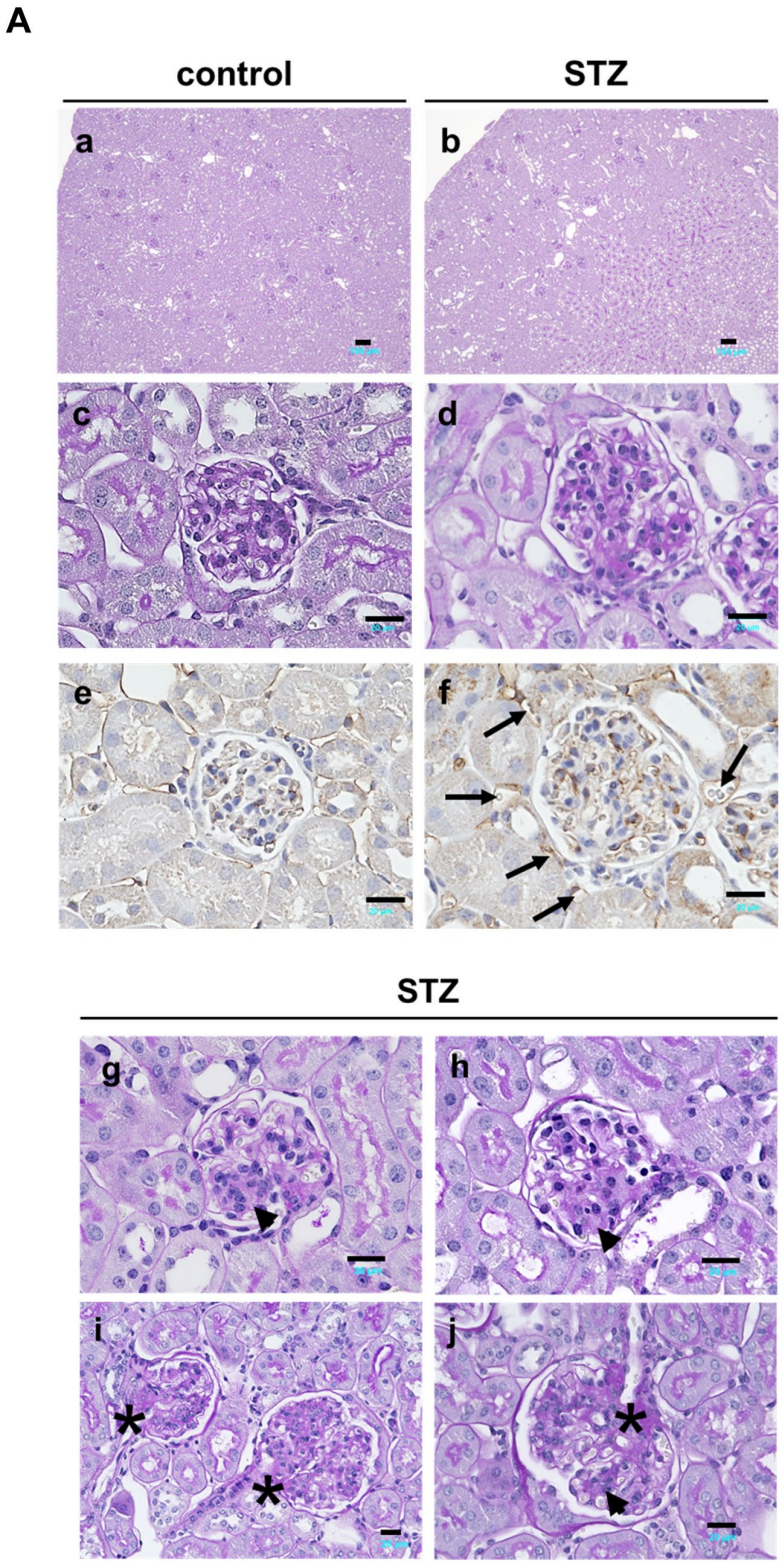

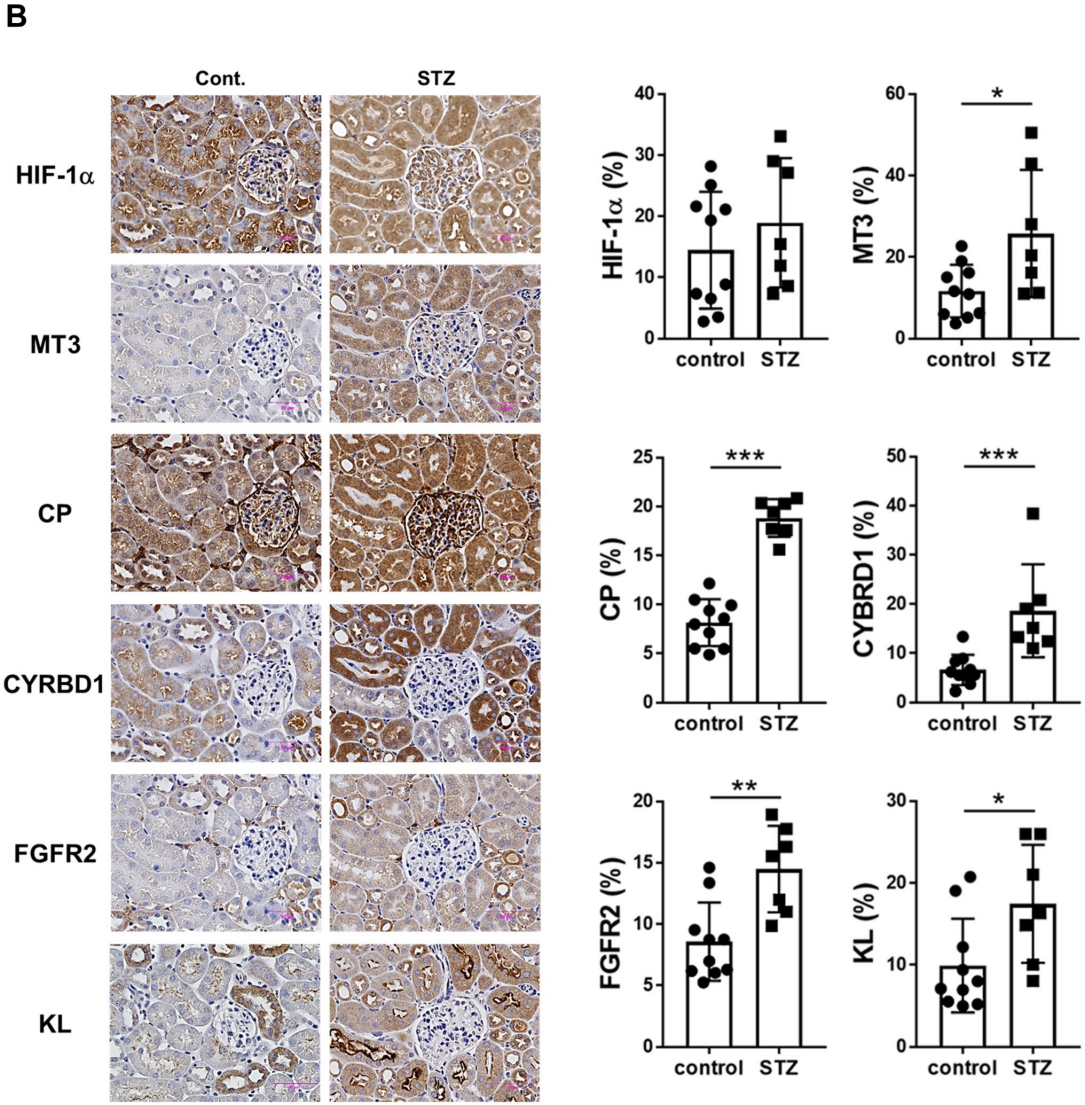

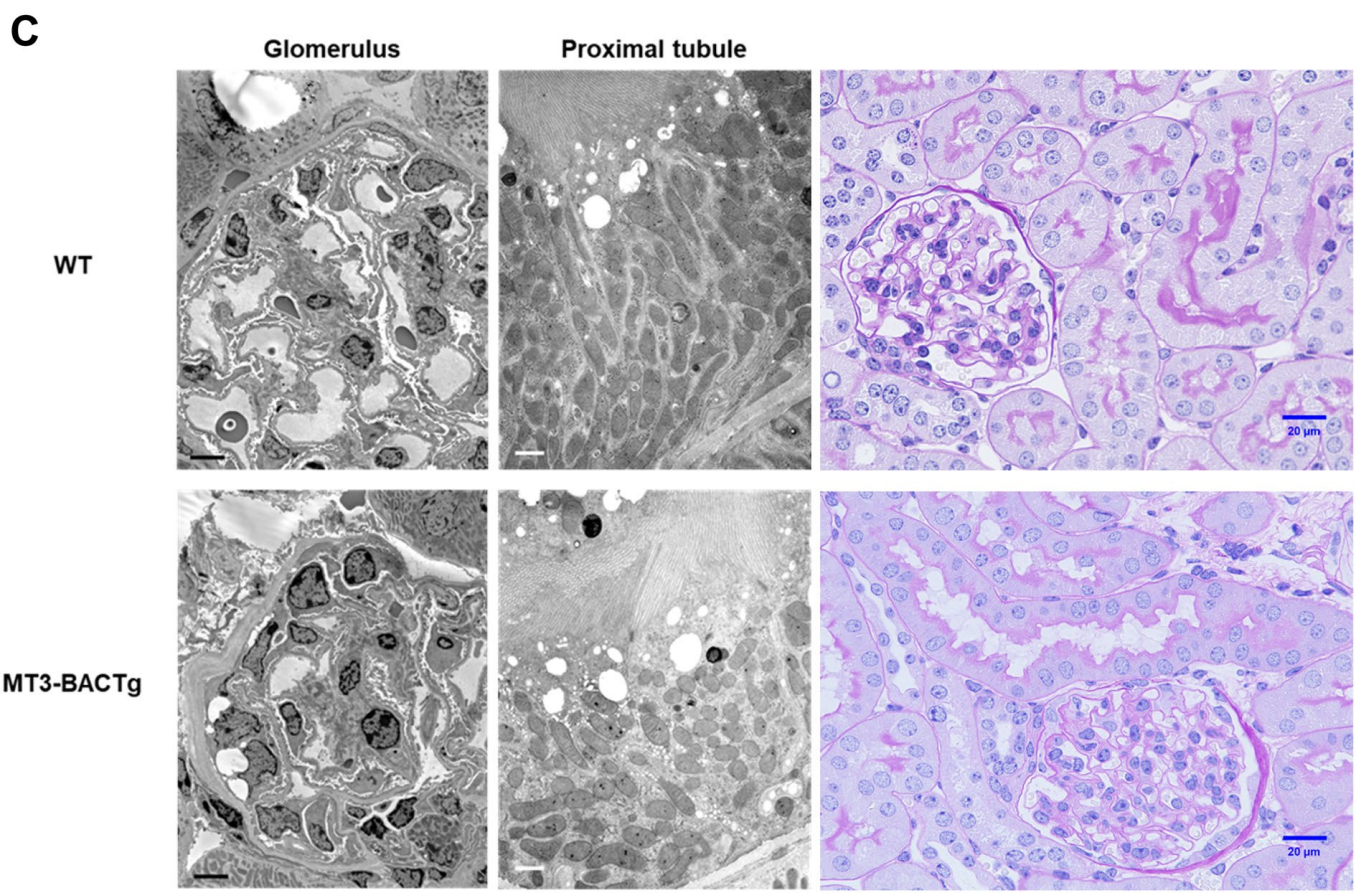
Histology of kidneys from humanized bacterial artificial chromosome transgenic mice (MT3-BACTg mice) with diabetic changes. **A** Compared with control MT3-BACTg mice (control, **a**), Periodic acid-Schiff (PAS) staining revealed that STZ-induced diabetic MT3-BACTg mice (STZ) at 15 weeks of age exhibited mesangial expansion and tubular dilation (**b**). Intriguingly, staining for the endothelial cell marker CD31 revealed peritubular capillary dilatation in STZ (**e**). In addition, PAS staining shows nodular glomerular sclerosis (black arrows) (**g**, **h**) and arteriolar and vascular pole hyalinosis (black asterisks) (**i**, **j**) in diabetic MT3-BACTg mice. Scale bars, 200 μm in lower magnified and 20 μm in higher magnified photomicrographs. **B** Compared with control mice, STZ-induced diabetic MT3-BACTg mice presented significantly increased protein expression of MT3 and its regulatory molecules. Statistical comparisons were performed via the Mann‒Whitney *U* test. *p < 0.05, **p < 0.01, ***p < 0.001. **C** Representative electronic microscopy images of the kidneys of streptozotocin-induced diabetic mice. Diabetes induced thickening of the glomerular basement, fusion of the podocyte foot processes, mitochondrial swelling, and a decrease in the number of mitochondrial cristae in both groups of eight-month-old mice. PAS staining presented more severe tubular injury in diabetic MT3-BACTg mice compared with those in wild-type (WT) mice. Scale bars: black, 5 μm; white, 1 μm, blue; 20 μm

Periodic acid-Schiff (PAS) staining revealed that STZ-induced diabetic MT3-BACTg mice exhibited mesangial expansion, nodular glomerular sclerosis, and proximal tubular injury, as shown by STZ-induced diabetic renal histological changes (Fig. 5A d, g, h, j). Intriguingly, MT3-BACTg mice presented dilated peritubular capillaries surrounding the glomeruli (Fig. 5A e, f). Diabetes increased the concomitant expression of the MT3, CP, CYRBD1, FGFR2, and KL proteins in renal tubules (Fig. 5B). Unexpectedly, no significant difference was observed in HIF-1α protein expression in control and diabetic group (Fig. 5B).

### MT3 aggravates diabetic renal injury associated with mitochondrial injury

To confirm the effects of MT3 on DN, we investigated the effects of MT3 on established STZ-induced DN via transmission electron microscopy (TEM) in wild-type littermates and MT3-BACTg mice at 8 months of age (Fig. 5C). Notably, the MT3-BACTg mice presented greater variations in mitochondrial size, the absence of basal infoldings, total disorientation of the mitochondria within the cell cytoplasm, and a decrease in the number of mitochondria compared with wild-type littermates (Fig. 5C), accompanied with more severe tubular injury shown in PAS staining. Thus, sustained higher level of MT3 might promote progression of DN.

### STZ-induced MT3-BACTg mice present increased renal expression of *MT3*, not *Mt3*, accompanied by increased *Cp, Cyrbd1, Fgfr2,* and* Kl*

**Fig. 6:**
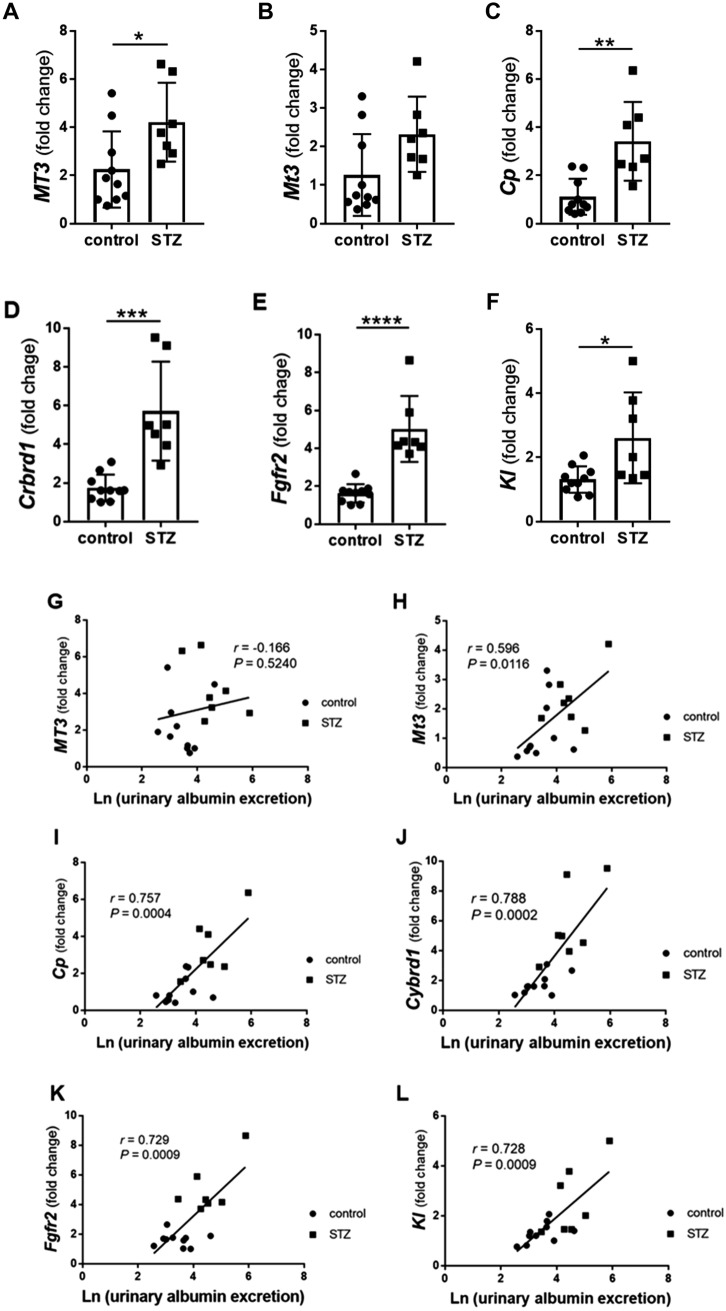

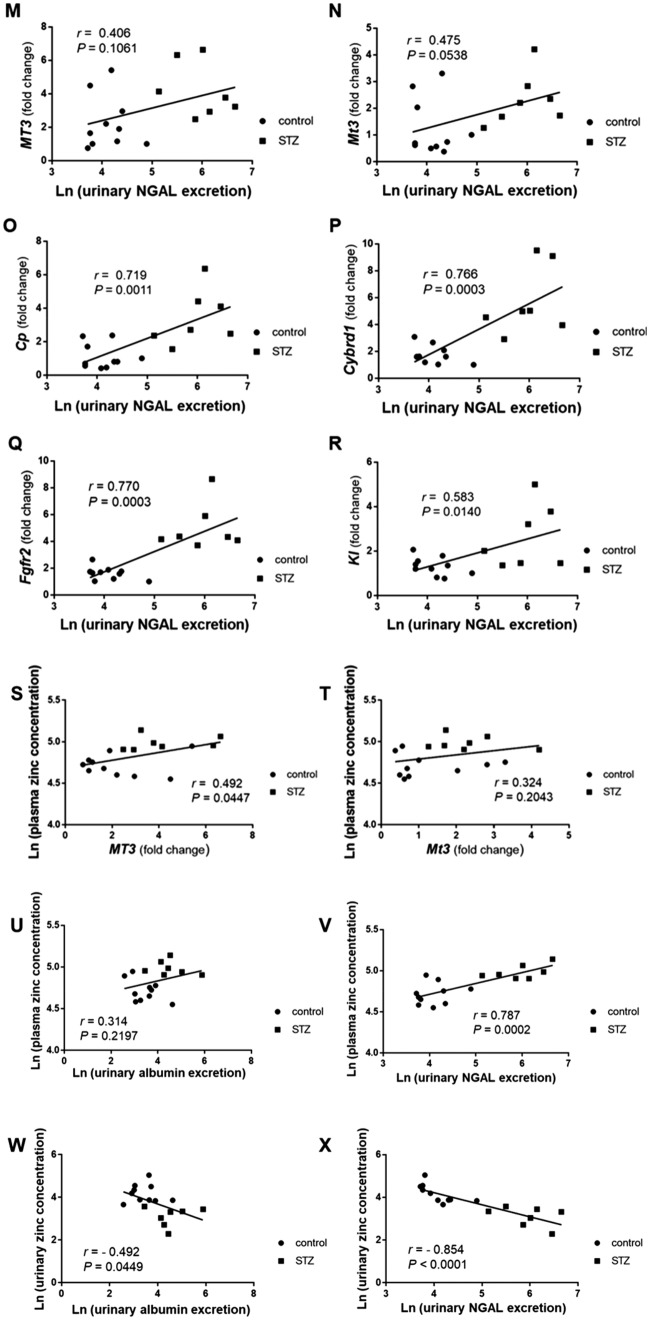
The expression of *MT3*, *Mt3*, *Cp*, *Crbrd1*, *Fgfr2*, and *Kl* in the cortex of kidneys from humanized bacterial artificial chromosome transgenic mice (MT3-BACTg mice) as determined via quantitative reverse transcriptase PCR (RT-PCR). **A** *MT3* expression was significantly increased in the streptozotocin (STZ)-induced diabetic group. **B** *Mt3* expression was not different between the control group and the STZ-induced diabetic group. Compared with control mice, STZ-induced diabetic mice presented greater expression of *Cp* **C**, *Crbrd1* **D**, *Fgfr2* **E,** and *Kl* **F**. Statistical comparisons were performed via Student’s *t* test. *p < 0.05, **p < 0.01, ***p < 0.001 and ****p < 0.0001. Urinary albumin excretion was statistically correlated with renal cortical *Mt3* **H**, *Cp* **I**, *Cyrbd1* **J**, *Fgfr2* **K,** and *Kl* **L** expression but not with *MT****3*** expression **G**. Urinary NGAL excretion was statistically correlated with renal cortical *Cp* **O**, *Cyrbd1* **P**, *Fgfr2* **Q,** and *Kl* **R** expression but not with *MT3* **M** or *Mt3* **N** expression. Plasma zinc levels were statistically correlated with renal cortical *MT3* **S** and urinary NGAL excretion **V** but not with *Mt3* **T** or urinary albumin excretion **U**. Urinary zinc levels were statistically correlated with urinary albumin excretion **W** and urinary NGAL excretion **X**. The relationships between two variables were analyzed via simple linear regression. *p* values < 0.05 were considered significant. Black circles: control MT3-BACTg mice (*n* = 10). Black squares: STZ-induced diabetic MT3-BACTg mice (*n* = 7)

Next, we analyzed gene expression in the renal cortex of MT3-BACTg mice via qRT-PCR. *MT3*, *Cp, Cybrd1, Fgfr2, and Kl* expression was significantly increased in the STZ group compared with the control group (Fig. 6A, C–F). *Mt3* expression did not significantly differ between the control group and the STZ group (*p* = 0.97) (Fig. 6B).

*MT3* expression was not correlated with urinary excretion of albumin (Fig. 6G) or NGAL (Fig. 6M). Moreover, *Mt3* expression was not correlated with urinary NGAL excretion (Fig. 6N), a marker of renal tubular injury. Nevertheless, *Cp, Cybrd1, Fgfr2,* and *Kl* expression was significantly correlated with urinary albumin excretion (Fig. 6H–L) and urinary NGAL excretion (Fig. 6O–R).

### Plasma zinc levels are correlated with renal expression of *MT *but not *Mt3*

To determine the effects of MT3 on zinc metabolism in STZ-induced diabetic mice, we evaluated the correlation between zinc levels and renal function in control and STZ-induced diabetic MT3-BACTg mice. Plasma zinc levels were correlated with renal *MT3* (*p* < 0.05, Fig. 6S) and urinary NGAL excretion (*p* < 0.001, Fig. 6V) but not with renal *Mt3* (*p* = 0.2043, Fig. 6T) or urinary albumin excretion (*p* = 0.2197, Fig. 6U). In contrast, urinary zinc levels were not correlated with *MT3* or *Mt3* expression (Supplementary Fig. 3). Urinary zinc levels were significantly correlated with urinary albumin excretion (*p* < 0.05, Fig. 6W) and urinary NGAL excretion (*p* < 0.0001, Fig. 6X).

### Aged MT3-BACTg mice present renal diabetic lesions, as observed in diabetic subjects

**Fig. 7:**
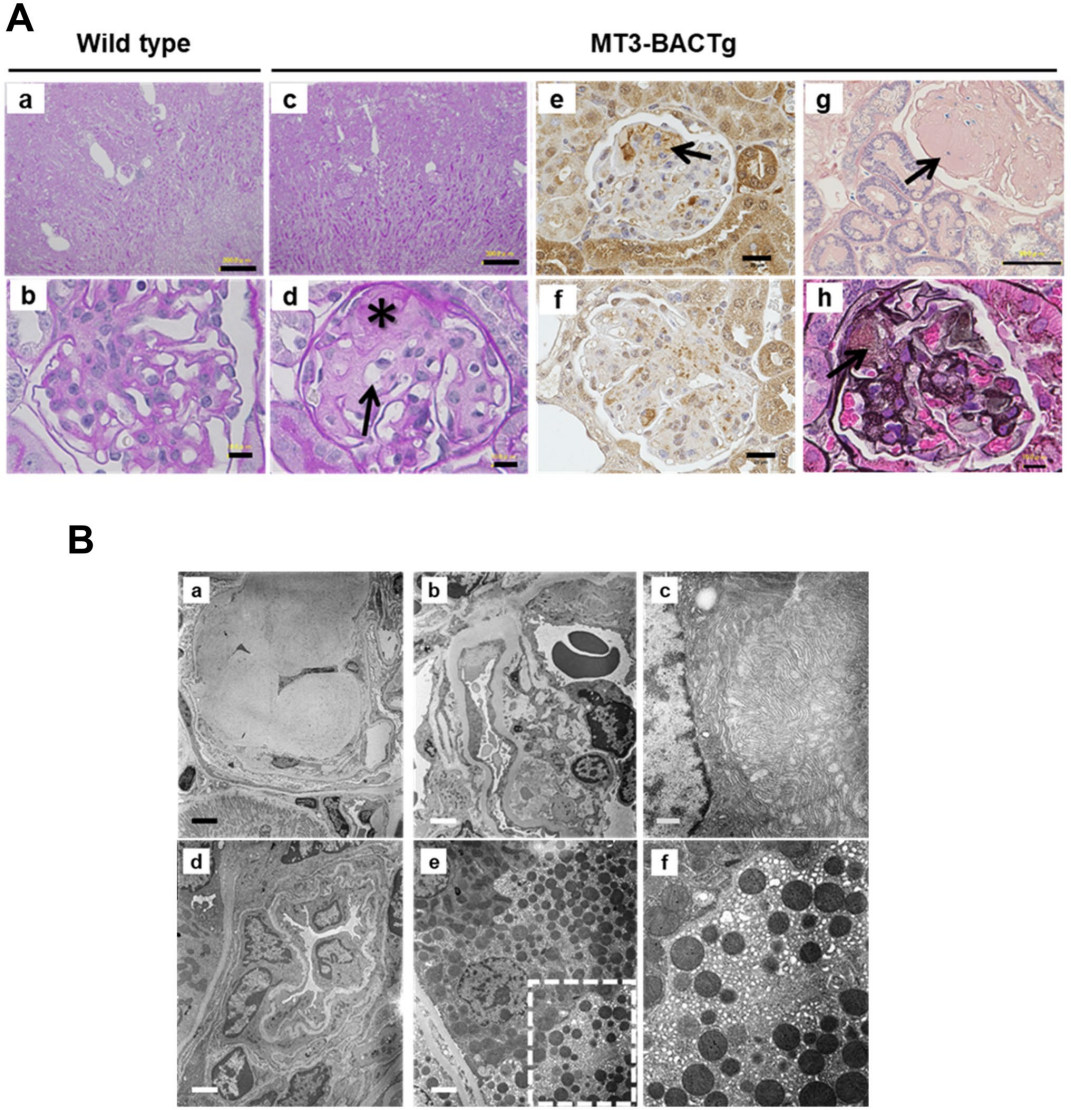
Aging induces severe kidney injury in MT3-BACTg mice. **A** PAS staining revealed that compared with diffuse mesangial expansion in the kidney of wild-type littermates (**a**, **b**), aged male MT3-BACTg mice with MT3 overexpression in renal tubules presented nodular glomerulosclerosis, such as Kimmelstiel–Wilson lesions (asterisk) and doughnut lesions (arrows) (**c**, **d**). Scale bars: 300 μm in a, c, 10 μm in b, d. MT3 immunopositive stains were present in the nodular lesion (**e**, arrow) and in the mesangial lesions (**f**). Scale bars: 20 μm. The glomerular nodules in MT3-BACTg mice were negative for PTAH (**g**) and positive for PAM (**h**, arrow). Scale bars: 50 μm in a, 10 μm in c. **B** Micrographs of MT3-BACTg glomeruli and proximal tubules at 2 years of age obtained via electron microscopy. **a** The mesangium was severely expanded with nodule formation. No electron-dense deposits were found. **b** Glomerular basement membranes (GBMs) were often severely thickened. **c** The number of endoplasmic reticula increased in the proximal tubules. **d** The swelling of endothelial cells caused narrowing of the capillary lumen. **e**, **f** The proximal tubules presented fewer abnormally shaped mitochondria, accompanied by the ubiquitous presence of the endoplasmic reticulum. **f** Magnified image of the decrease in the number and size of mitochondria in aged MT3-BACTg proximal tubules. Representative transmission electron micrograph. Scale bars: black, 5 μm; white, 2 μm; gray, 500 nm. PAS, periodic acid–Schiff. PTAH, phosphotungstic acid-hematoxylin. PAM, periodic acid methenamine

Despite its role as an inducer of KL, the MT3 transgene showed no antiaging effects on MT3-BACTg mice (Supplementary Fig.  4 A). To confirm the effects of the MT3 transgene on aged kidneys without diabetes, we studied MT3-BACTg mice at age 2 years. Intriguingly, aged MT3-BACTg mice presented no significant difference in glucose metabolism compared to aged wild-type mice (Supplementary Fig. 4B, C), which was accompanied by glomerular nodules such as those associated with DN (Fig. 7A). The glomerular nodules in MT3-BACTg mice were negative for phosphotungstic acid-hematoxylin stain (PTAH), which indicates fibrin deposition (Fig. 7A). Periodic acid methenamine silver stain (PAM) presented mesangiolytic nodular lesions [23] in the kidneys of aged MT3BACTg mice (Fig. 7A). The mesangium was severely expanded with nodule formation, but no electron-dense deposits were observed in the glomerular nodules (Fig. 7B, a). The number of mesh-like endoplasmic reticula was increased in the proximal tubules, indicating cellular injury [24] (Fig. 7B, c). In addition, TEM revealed the obstruction of downstream peritubular capillaries (Fig. 7B, d), which leads to glomerular hypertension. Continuous MT3 expression might present more severe DN via retrograde glomerular hypertension.

### MT3 inhibited HIF-1α expression in renal proximal tubular cells in vitro and in vivo

**Fig. 8:**
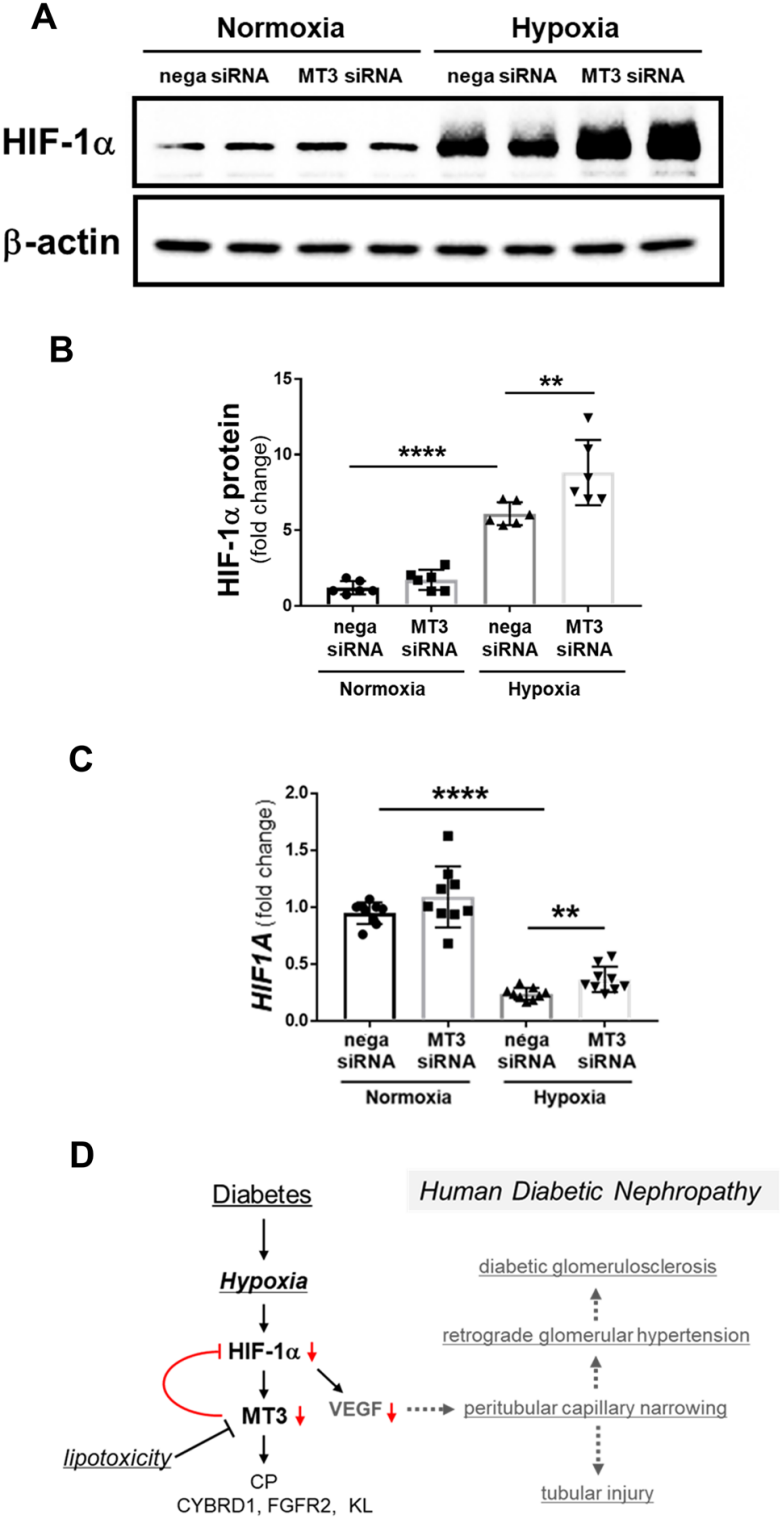

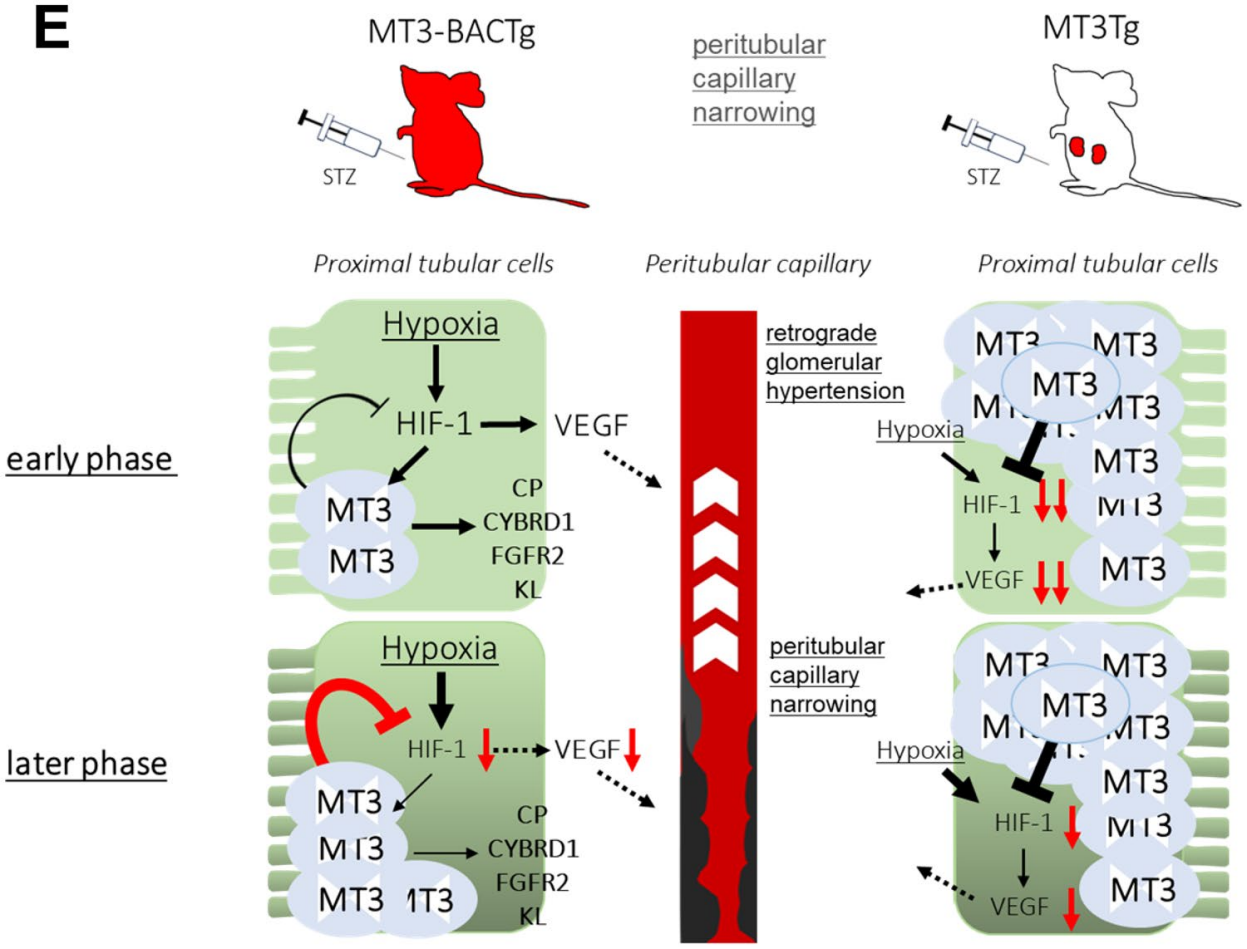
Human MT3 inhibits hypoxia-induced HIF-1α protein expression in HRPTECs. **A** MT3 siRNA increased hypoxia-induced HIF-1α protein expression. **B** Densitometric analysis showed that hypoxia significantly induced HIF-1α protein expression compared with that in a negative control (nega) siRNA in normoxia and that MT3 siRNA significantly increased hypoxia-induced HIF-1α protein expression to that of a negative control (nega) siRNA in hypoxia. **C** Hypoxia significantly inhibited *HIF1A* compared with that in normoxia. MT3siRNA significantly increased *HIF1A* in normoxia and hypoxia compared with that in the control. One-way repeated-measures ANOVA with Bonferroni's multiple comparison post hoc tests, **p < 0.01, ****p < 0.0001. **D** Interplay between MT3 and HIF-1α in human diabetic nephropathy. Chronic hypoxia-induced MT3 expression might inhibit HIF-1α/VEGF expression in renal proximal tubules, resulting in peritubular capillary injury which causes retrograde glomerular hypertension. **E** HIF-1α expressions were inversely correlated with MT3 expressions in renal proximal tubular cells in both MT3-BACTg and MT3Tg mice

The rat remnant kidney model identified the characteristic peritubular capillary rarefaction along with decreased interstitial VEGF expression. VEGF is one of HIF-1-targeted genes. To clarify the mechanism which induced the obstruction of peritubular capillary observed in TEM study, we studied the effects of MT3 on HIF-1α expression using HRPTECs. MT3siRNA significantly increased hypoxia-induced HIF-1α proteins accompanied with *HIF1A* increment (Fig. 8A, B and C). To validate the effects of MT3 on HIF-1α protein in vivo, we used proximal tubule-specific overexpressed human *MT3* transgenic mice (MT3Tg). We found no overlap protein expression of HIF-1α and MT3 in the proximal tubules of diabetic MT3Tg (Supplementary Fig. 5). Overexpression of MT3 also failed to prevent diabetic nephropathy in STZ-induced MT3Tg mice as seen in MT3-BACTg mice (Supplementary Table 7).

### Relationship between the GFR and *MT3* gene expression

To explore the clinical significance of MT3 in DN, we accessed the Nephroseq database (https://www.nephroseq.org/) and obtained relevant microarray data from the ‘Woroniecka Diabetes Tubint’, which includes 22 human diabetic kidney disease samples. In DN patients, correlation analysis revealed a positive correlation between MT3 mRNA expression in the renal tubulointerstitium and the glomerular filtration rate (GFR) (*r* = 0.650, *p* = 0.001; Supplementary Fig. 6), revealing that MT3 may increase in the early stage of DN accompanied with relative hypoxia, which is revealed by increased oxygen consumption.

## Discussion

In this study, we demonstrated that hypoxia induces *MT3* expression associated with increased expression of *CP, CYBRD1, FGFR2,* and *KL* in HRPTECs. In addition, STZ-induced diabetic MT3-BACTg mice at age of 15 weeks presented increased expression of these factors in renal tubules and increased levels of plasma and urinary zinc levels.

Through qRT-PCR, we investigated the top three MT3 siRNA-downregulated genes, *CP, CYRBD1,* and *FGFR2* in HRPTECs. We found lack of reproducibility regarding hypoxia-induced the expressions of *CYBRD1* and *FGFR2* in HRPTECs, which might be due to the limited effects of hypoxia on *CYBRD1* and *FGFR2* compared to *CP* and the variations of individual lots of HRPTECs*.* A recent study demonstrated that ferroptosis was involved in diabetic kidney disease progression, and CP acted as a central regulator of the induction of ferroptosis [18]. CP is known as a copper transporter and the antioxidant system [19]. Serum and urinary CP levels increase in DN patients [20]. Urinary CP levels are positively associated with proximal tubular injury [18]. Our study clarified the mechanism by which diabetes increased CP levels via hypoxia-induced MT3 in DN patients.

Previous studies demonstrated that high glucose per se fails to induce renal hypoxia in vivo [21] and HIF-1α in HRPTECs [22]. In line with these results, PA, not high glucose inhibited the expressions of these molecules, suggesting that lipotoxicity, not glucotoxicity, plays an important role via changing expression of these factors in DN. Free fatty acids as PA are the only complex I–ubiquinone-junction site inhibitors demonstrating the titration pattern, which is fairly in line with the presence of two piericidin (a natural complex I inhibitor)-binding sites [23, 24]. Consistent with the results of mitochondrial complex inhibitors (rotenone, antimycin A) on hypoxia-induced the expressions of *MT3* and HIF-1α protein in HRPTECs as described in this study and the previous study [22], PA might suppress the expression of hypoxia-induced *MT3* and HIF-1 α protein via the inhibition of mitochondrial complex I.

Hypoxia did not induce *HIF1A* in HRPTECs, consistent with the previous studies [22, 25, 26]. Interestingly, hypoxia induced *MT3* expression in HRPTECs, not *Mt3* expression in mouse proximal tubular cells (mProx). Previous studies demonstrated that the promoter regions of mouse *Mt3* and human *MT3* have little direct sequence identity [27, 28]. Hence, the JASPAR analysis revealed fewer putative site(s) for HIF1A in the promoter region of the mouse *Mt3* gene than human *MT3*. These different sequences in their promoter regions between *MT3* and *Mt3* might be involved in their different expression in the kidneys of STZ-induced diabetic MT3-BACTg mice.

HIF-1α siRNA and Nrf2 siRNA decreased hypoxia-induced *MT3* expression in HRPTECs. Nrf2 acts against oxidative stress, including ROS [29, 30]. However, the antioxidant NAC failed to inhibit hypoxia-induced *MT3* in HRPTECs, indicating that ROS might not be involved in hypoxia-induced *MT3* expression. A previous study demonstrated that Nrf2 mediates hypoxia-inducible HIF-1α activation using *Nrf2*-sufficient and *Nrf2*-deficient primary murine renal tubular epithelial cells [31]. Thus, *MT3* expression mainly depends on HIF-1α.

Compared with control MT3-BACTg mice, STZ-induced diabetic MT3-BACTg mice presented significantly higher MT3, CP, CYRBD1, FGFR2, and KL mRNA and protein levels in the kidney. These MT3-regulated genes, *Cp*, *Cybrd1*, *Fgfr2* and *Kl*, presented significant correlations with urinary markers. However, *MT3* expression was not correlated with urinary albumin or NGAL excretion. MT1/MT2 [32], albumin [33] and NGAL [34] are taken up by the multiligand endocytic receptor megalin [35] on the renal proximal tubular cells. In addition, MT3, as well as MT1 and MT2, possesses the megalin-interacting fragment SCKKSCC (Serine-Cysteine-Lysine-Lysine-Serine-Cysteine-Cysteine) [36] in its amino acid sequences [37]. Therefore, excessive MT3 might compete with albumin and NGAL for megalin-mediated reabsorption in the renal proximal tubular cells. Furthermore, as oxidative stress increases megalin expression in the renal proximal tubular cells in STZ-induced diabetic rats [38], antioxidant MT3 may decrease megalin expression, leading to increased urinary levels of albumin and NGAL.

Additionally, urinary and plasma zinc levels were increased in STZ-induced diabetic MT3-BACTg mice. A previous study demonstrated that STZ-induced diabetes in male Wistar rats significantly increased renal zinc levels [39]. Higher plasma zinc levels and increased urinary zinc loss were reported in women with insulin-dependent diabetes mellitus (IDDM) [40]. Hyperzincuria in IDDM is correlated with urinary protein excretion [40], which is in line with the correlation of the urinary zinc level with urinary albumin and NGAL excretion observed in the current study.

The MT3 transgene exacerbated diabetic pathological changes, especially tubular mitochondria injury, as observed via TEM in STZ-induced wild-type mice and MT3-BACTg mice at age 8 months. MT3, similar to MT1, inhibits respiratory inhibition in liver mitochondria [41]. Mitochondria respiratory inhibition results in increased intracellular oxygen concentrations and decreased mitochondrial ROS production as described in nitric oxide [42] and metformin [22]. The short period of respiratory inhibition by MT3 might be renoprotective for DN, but its persisting effects might lead to cellular anoxia. Thus, sustained hyperglycemia might induce renal mitochondria injury via hypoxia-induced MT3. Unexpectedly, aged MT3-BACTg mice presented glomerular nodules without diabetes. MT3-BACTg mice show obstruction of downstream peritubular capillaries. The rat remnant kidney model identified the characteristic peritubular capillary rarefaction along with decreased interstitial VEGF expression [43]. Previous study demonstrated renal tubular epithelial cells-derived small extracellular vesicle-VEGFA promotes peritubular capillary repair in ischemic kidney injury [44]. As *VEGFA* is one of HIF-target genes [45], we studied the effect of MT3 on HIF-1α expression. MT3 siRNA augmented HIF-1α protein and *HIF1A* expression in HRPTECs. Chowdhury et al. showed that MT3 suppresses HIF-1α stabilization and glycolysis in human macrophages [46]. In accordance with the action in macrophages, MT3-lowering increased hypoxia-induced HIF-1α protein expression accompanied with *HIF1A* increment in HRPTECs. Then, chronic hypoxia- or oxidative stress-induced MT3 expression could decrease HIF1α/VEGF expression in renal proximal tubules, resulting in peritubular capillary injury which causes retrograde glomerular hypertension (Fig. 8D). Furthermore, in STZ-induced MT3Tg mice, proximal tubules-specific overexpression of MT3 surprisingly decreased the expression of HIF-1α protein and failed to ameliorate DN as shown in MT3-BACTg mice. Thus, the interplay between MT3 and HIF-1α might be involved in no significant difference of HIF-1α protein in control and STZ-induced diabetic MT3-BACTg mice (Fig. 8E).

This study has several limitations. First, previous studies also indicated that hypoxia induces MT3 mRNA and protein expression in normal human astrocytes (NHA6700 cells) [47] and MT3 mRNA in primary human adipocyte cell culture [48]. However, the precise mechanism how hypoxia induces MT3 expression in human cells remains obscure. Second, the relationship between HIF-1α and MT3 also need further research to determine how MT3 suppresses HIF-1α mRNA and protein expression. Third, clinical studies examining the dynamic changes of MT3 in blood, urine and kidney tissues of patients with DN will be vital in translating our results to patient outcomes. Our future studies will focus the mechanisms by which MT3 regulates many genes including HIF-1α and metabolism in renal proximal tubular cells.

Our work highlights the central role of MT3 as a regulator of hypoxia-induced diabetic tubular injury associated with the regulation of biogenic metallic elements, e.g., zinc [49]. In addition, our study identifies MT3 as a novel therapeutic target and biomarker and provides a new humanized mouse model for DN.

## Supplementary Information

Below is the link to the electronic supplementary material.

Supplementary file1 (DOCX 2336 KB)
