# Supplementary material for: Role of metallothionein 3 in diabetic nephropathy via interplay with HIF-1α
Source: Diabetol Int. 2025 Aug 19;16(4):779–800. doi: 10.1007/s13340-025-00840-y (PMC12532810; doi:10.1007/s13340-025-00840-y)
Supplement: Supplementary file 1 — Supplementary file1 (DOCX 2336 KB) [file 13340_2025_840_MOESM1_ESM.docx]

**Supplementary data**

**Role of Metallothionein 3 in Diabetic Nephropathy via Interplay with HIF-1α**

**Yuri Takiyama^1^, Yumi Takiyama^1^, Takao Takiyama^1^, Ryoichi Bessho1^1^, Hiroya Kitsunai^1^, Akira Takasawa^3^, Hiroshi Nomoto^1^.**

From the ^1^Division of Endocrinology, Metabolism and Rheumatology, Department of Medicine, Asahikawa Medical University.

^2^Division of Tumor Pathology, Department of Pathology, Asahikawa Medical University.

Address all correspondence to: Yumi Takiyama, MD, PhD.

E-mail address: taka0716@asahikawa-med.ac.jp

Phone: +81-166-68-2454

**Supplementary methods.**

**Cell culture**

HPRTECs were purchased as once- or twice-passaged tubular cells from Lonza Walkersville, Inc. (Walkersville, MD). The cells were grown in renal epithelial cell growth medium (REGM; Lonza) in collagen type 1-coated dishes at 37 °C in an incubator containing 5% CO_2_ and 95% humidified air as previously described [1]. The cells were exposed to reagents under normoxic (21% O_2_) or hypoxic (1% O_2_) conditions for 24 h before being harvested for experiments as previously described [2]. To study the effects of glucose and fatty acids, the cells were incubated in medium containing 5.5 mM (low) glucose, 25.5 mM (high) glucose, saturated free fatty acid (FFA) palmitic acid (PA; 150 μM) or unsaturated FFA oleic acid (150 μM). Saturated FFA such as PA have cytotoxic effects, whereas unsaturated FFA inhibit palmitate-induced renal tubular damages [3]. We perform the experiment using FFAs at the concentration and incubation time which was proven to present their effects on proximal tubular epithelial cells in the previous studies [3] [4]. PA (Sigma‒Aldrich, St. Louis, MO) and oleic acid (Sigma‒Aldrich) were dissolved in 50% ethanol heated to 60 °C and mixed with a 12.5% free fatty acid (FFA)-free bovine serum albumin (BSA) (Sigma‒Aldrich) solution in low-glucose Dulbecco’s modified Eagle’s medium (DMEM). A mixture of 50% ethanol and FFA-free BSA was used as a control [3] [5].

**Small interfering RNA transfection**

Lipofectamine RNAiMAX transfection reagent (Thermo Fisher Scientific Inc., Waltham, MA) was mixed with small interfering RNAs (siRNAs) (Dharmacon, ON-TARGETplus siRNA, Horizon Discovery, Cambridge, U.K., Supplementary Table 1) in Opti-MEM reduced serum medium (Gibco, Carlsbad, CA) according to the manufacturer’s instructions. The cells were incubated for 24 h and exposed to each condition.

**Conventional reverse transcriptase PCR**

Total RNA was extracted from HRPTECs with an RNeasy Mini Kit (Qiagen, Valencia, CA) according to the manufacturer's instructions. *MT3* cDNA amplification was performed with the Expand High Fidelity PCR system (Boehringer Mannheim, Mannheim, Germany) in a final volume of 25 µl containing one-fifth of reverse transcriptase (RT) products, 10 pmol of sense and antisense primers (2 sets of primers; Supplementary Table 2), and 1.75 units of Taq DNA polymerase as described previously [1]. Loading dye and TE buffer were added to each PCR product, which was subsequently loaded onto 1% agarose gels.

**Microarray analysis**

To analyze the gene expression levels in the renal tubular cells of rats at 17 weeks of age and HRPTECs, we used the GeneChip Rat Gene 1.0 ST Array (Thermo Fisher) and Human Gene 1.0 ST Array (Thermo Fisher) respectively. The RNA was quantified with an ND-1000 spectrophotometer (NanoDrop Technologies, Wilmington, DE), and its purity and quality were determined with a Bioanalyzer 2100 (Agilent Technologies, Santa Clara, CA). Only RNA samples with an integrity number >8.0 were used. Fifty nanograms of total RNA was processed with a GeneChip WT Terminal Labeling Kit (Affymetrix, Santa Clara, CA) and hybridized to Affymetrix Rat Gene 1.0 ST gene chips and Affymetrix Human Gene 1.0 ST gene chips respectively. Chips were stained and washed with an Affymetrix GeneChip Fluidics Station 450 and scanned with a GeneChip Scanner 3000 7G. Microarray data analysis was performed via GeneSpring GX version 11.5.1 (Agilent Technologies). When the value of a probe was lower than the 20th percentile value in any sample, the probe was excluded. Since linear fold changes span positive and negative spaces, the threshold for absolute linear fold change values was set to > 1.0 to cover both sides. All procedures were performed according to the manufacturer’s instructions.

**Quantitative RT‒PCR**

Total RNA was extracted from HRPTECs and the left kidney cortex from mice in each group through the use of an RNeasy Mini Kit (Qiagen) according to the manufacturer's instructions. cDNA synthesis was performed with a high-capacity cDNA Reverse Transcription Kit with RNase Inhibitor (Applied Biosystems, Carlsbad, CA). Each cDNA sample was analyzed for gene expression via quantitative RT–PCR (qRT–PCR) via a fluorescent TaqMan 57-nuclease assay and a sequence detection system (Prism 7300, Applied Biosystems). TaqMan real-time PCR was performed using 2× TaqMan Master Mix and 20× assay-on-demand TaqMan primers and probes (Applied Biosystems). The analysis was performed with ABI Prism 7300 SDS software (version 1.4.1, Applied Biosystems, https://www.thermofisher.com/jp/ja/home/technical-resources/software-downloads/applied-biosystems-7300-real-time-pcr-system.html). Unlabeled specific primers were purchased from Applied Biosystems to detect the genes shown in Supplemental Table 3. For quantitative analysis, the cDNA content of each sample was normalized to the level of the housekeeping gene RPLP0 via the comparative C_T_ method.

**Animal models**

At 15 weeks of age, the body weight, systolic blood pressure (SBP), food intake, water intake, fasting blood glucose (FBG), glycated hemoglobin (HbA1c), and plasma cystatin C level (Mouse/Rat Cystatin C Quantikine ELISA Kit, Bio-Techne R&D Systems, Minneapolis, MN) and zinc level (Zinc Assay Kit, Metallogenics Co., Ltd., Chiba, Japan) of the MT3-BACTg mice and MT3Tg mice were measured. Glucose tolerance tests (GTT) were performed as we previously described [6]. We evaluated the urinary markers, including urinary volume, urinary albumin excretion (Mouse Urine Albumin ELISA Kit, Exocell, a brand of Ethos Biosciences, Inc., Logan Township, NJ), urinary neutrophil gelatinase-associated lipocalin (NGAL) (Mouse Lipocain-2 Quantikine ELISA Kit, Bio-Techne R&D Systems) and urinary zinc levels, for 24 h by placing mice in metabolic cages. The removed kidneys were used for histological and biochemical studies, including transmission electron microscopy (TEM), immunohistochemical staining of mouse tissue sections.

**Lifespan of the mice**

Male mice were inspected daily for health issues, and any deaths were recorded. Animals showing significant signs of morbidity were euthanized for humane reasons and were used for lifespan analysis since they were deemed to live to their full lifespan. No mice were censored from the analysis. Lifespan was analyzed via Kaplan–Meier survival curves, and *p* values were calculated via the log-rank (Mantel‒Cox) test via GraphPad Prism. n = 6 mice per group.

**Immunohistochemistry**

Immunohistochemical staining was performed as previously described [7] [8]. The tissue sections were incubated with the primary antibodies listed in Supplementary Table 4. The stained sections were observed and visualized with a light microscope system (BZ-X810; Keyence Co., Osaka, Japan). Immunohistochemical images were quantified with Fiji ImageJ (US National Institutes of Health, Bethesda, MD, USA, ImageJ.net/Fiji, v.1.52) as described in a previous study [8].

**Electron microscopy**

Parts of the removed kidneys were cut into small tissue blocks (1 mm^3^) and fixed with 2.0% glutaraldehyde and 2.0% paraformaldehyde in 0.1 mol/l phosphate buffer at 4 °C. After fixation with 2% osmium tetroxide, the tissues were dehydrated in a series of graded ethanol solutions, and the ethanol was then substituted with propylene oxide. The tissues were then embedded in epoxy resin. Ultrathin sections were double-stained with uranyl acetate and lead. The sections were examined under an electron microscope (JEM1200EX, JEOL, Tokyo, Japan) at 80 kV.

**Clinical analysis**

The Nephroseq v5 database (http://v5.nephroseq.org) is a comprehensive information platform for evaluating the correlation between gene expression levels and the clinical characteristics of patients with kidney diseases. To explore the correlation between the expression of *MT3* and clinical features, we mined the Nephroseq v5 database

| **Supplementary Table 1. Catalog numbers and sequences of small interfering RNAs (siRNAs) used in this study.** |
| --- |

|  |  |
| --- | --- |
|  | |
|  | |
|  | |

The negative control was a pool of four siRNAs designed for minimal targeting of human genes (ON-TARGETplus nontargeting control pool). All siRNAs used were Dharmacon^TM^ ON-TARGETplus^TM^ siRNAs (Horizon Discovery, a PerkinElmer company, Cambridge, GB).

**Supplementary Table 2. Primer sequences, conditions and product sizes for conventional reverse transcriptase PCR (RT‒PCR).**


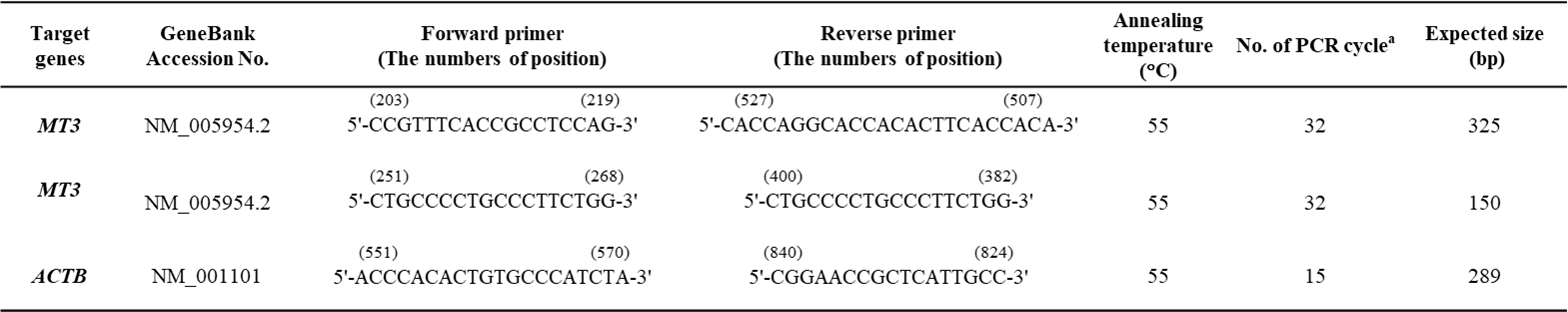


^a^ Numbers of cycles were determined to amplify both products logarithmically and in relatively similar amounts.

**Supplementary Table 3. qRT-PCR primers purchased from Applied Biosystems.**

| **Supplementary Table 4. Primary antibodies for immunohistochemistry.** | |  |  |  |
| --- | --- | --- | --- | --- |
|  |  |  |  |  |
| **Antibody** | **company** | **Catalog number** | **Application** | **Dilution** |
| HIF-1α | Novus Biochemicals | NB100-479 | IHC | 1/100 |
| Metallothionein 3 | Santa Cruz Biotechnology | sc-293488 | IHC | 1/50 |
| Ceruloplasmin | proteintech | 66456-1-Ig | IHC | 1/400 |
| Cytochrome b reductase 1 | proteintech | 26735-1-AP | IHC | 1/800 |
| Fibroblast growth factor receptor 2 | proteintech | 13042-1-AP | IHC | 1/400 |
| Klotho | proteintech | 28100-1-AP | IHC | 1/100 |
| CD31 | abcam | ab28364 | IHC | 1/50 |

IHC; Immunohistochemistry

**Supplementary Table 5. The JASPAR results for hypoxia response elements (HREs) in the promoters of *MT3***

**Supplementary Table 6. The JASPAR results for hypoxia response elements (HREs) in the promoter of *Mt3***

**Supplementary Table 7. Laboratory data of control and streptozotocin (STZ)-induced diabetic MT3-BACTg and MT3Tg at 15 weeks of age.**

| **Laboratory data** | **MT3-BACTg** | | | **MT3Tg** | | |
| --- | --- | --- | --- | --- | --- | --- |
|  | **Control (n=10)** | **Diabetes (n=7)** | ***P values*** | **Control (n=7)** | **Diabetes (n=6)** | ***P values*** |
| **Fasting blood glucose** (mg/dL) | 116.7 ± 42.4 | 326.6 ± 102.5 | <0.0001 | 114.7 ± 27.0 | 261.3 ± 108.8 | <0.01 |
| **HbA1c** (mmol/mol) | 21.3 ± 1.25 | 82.43 ± 16.38 | <0.0001 | 21.26 ± 2.14 | 60.00 ± 14.42 | <0.0001 |
| **HbA1c** (%) | 4.13 ± 0.13 | 9.28 ± 1.51 | <0.0001 | 4.11 ± 0.19 | 7.63 ± 1.31 | <0.0001 |
| **Body weight** (g) | 24.35 ± 2.03 | 20.77 ± 1.29 | <0.001 | 24.09 ± 3.39 | 20.10 ± 1.66 | <0.05 |
| **Systolic blood pressure** (mmHg) | 66.7 ± 7.41 | 77.86 ± 14.17 | 0.0504 | 77.43 ± 12.01 | 83.33 ± 7.81 | 0.084 |
| **Mean blood pressure** (mmHg) | 42.70 ± 6.50 | 49.43 ± 12.97 | 0.0632 | 49.14 ± 7.97 | 53.83 ± 4.49 | 0.2289 |
| **Plasma cystatin C** (ng/mL) | 478.4 ± 53.21 | 402.5 ± 43.28 | <0.01 | 489.61 ± 71.54 | 451.78 ± 103.33 | 0.4532 |
| **Plasma zinc** (mg/dL) | 112.6 ± 15.17 | 146.7 ± 13.23 | <0.001 | 106.26 ± 25.32 | 188.22 ± 88.72 | <0.05 |
| **Urinary volume** (mL/day) | 1.09 ± 0.54 | 10.31 ± 6.98 | <0.0001 | 0.87 ± 0.29 | 7.43 ± 5.74 | <0.05 |
| **Urinary albumin excretion** (mg/day) | 32.04 (19.99-43.55) | 85.28 (62.63-153.4) | <0.01 | 15.54 (12.65-20.39) | 73.8 (53.49-102.9) | <0.0001 |
| **Urinary NGAL excretion** (ng/day) | 54.95 (43.09-133.2) | 408.4 (244.6-641.4) | <0.001 | 47.08 (38.81-56.77) | 241.6 (87.13-740.8) | <0.01 |
| **Urinary zinc** (mg/day) | 0.67 ± 0.23 | 2.11 ± 1.38 | <0.01 | 0.73 ± 0.32 | 0.64 ± 0.56 | 0.7301 |

The data are presented as the means ± standard deviations (SDs) for normally distributed variables and medians (25th–75th interquartile range) for skewed variables unless otherwise indicated.


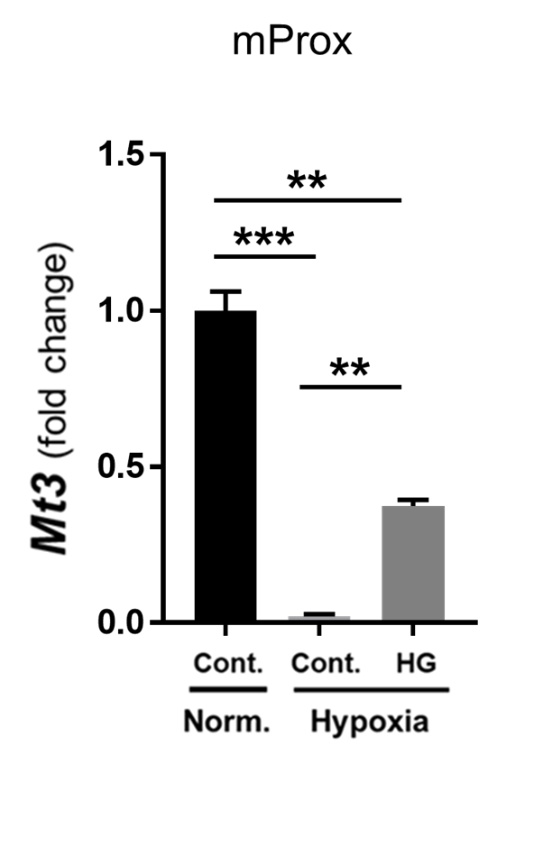
**Supplementary Fig. 1.**

**Supplementary Fig. 1. Hypoxia inhibits *Mt3* expression in murine proximal tubular (mProx) cells. mProx Cell Culture**

Immortalized murine proximal tubular (mProx) cells were kindly provided by Takeshi Sugaya, PhD [9]. mProx cells were cultured in DMEM supplemented with 10% heat-inactivated FBS, 100 U/ml penicillin, and 100 μg/ml streptomycin. Cultured cells from passages 4–30 were used for the experiments. Subconfluent cells were made quiescent by incubation with 0.1% FBS-DMEM for 24 h. Quiescent cells were incubated with 25.5 mM D-glucose under hypoxic conditions (1% O_2_) overnight. After incubation, the cells were harvested for qRT‒PCR. In contrast to human renal proximal tubular epithelial cells (HRPTECs), hypoxia inhibited *Mt3* mRNA compared with the control (Cont.) under normoxia (Norm.) High glucose (25.5 mM D-glucose; HG) conditions significantly and partially restored the hypoxia-induced inhibitory effect on *Mt3* expression in mProx cells. Statistical analysis was performed via ANOVA and Bonferroni post hoc correction. The data represent the means ± SDs. ****p*<0.001, ***p*<0.01.

**Supplementary Fig. 2.**


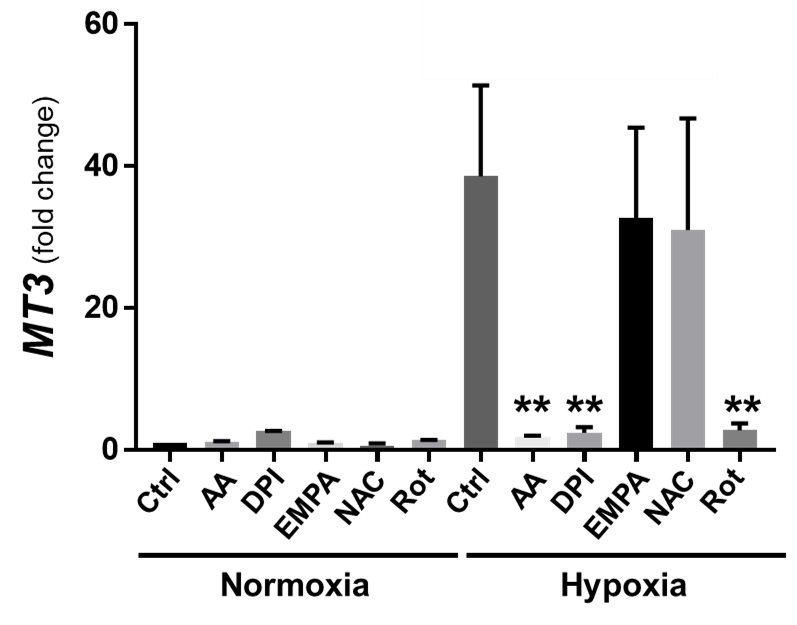


**Supplementary Fig. 2. Hypoxia*-*induced *MT3* expression *is* dependent on the mitochondrial respiratory system in human renal proximal tubular epithelial cells (HRPTECs).**

The antioxidant N-acetyl-cysteine (1 mM; NAC) failed to restore hypoxia-induced *MT3* expression, whereas mitochondrial respiratory inhibitors (rotenone 1 μM; Rot, antimycin A 1 μM: AA) and NADPH oxidase (NOX) inhibitors (diphenylene iodonium 10μM; DPI) abolished the stimulatory effect of hypoxia on *MT3* expression, indicating that hypoxia-induced *MT3* expression is dependent on the mitochondrial respiratory system or NOX. The SGLT2 inhibitor which is strongly recommended for use in people with type 2 diabetes and chronic kidney disease, empagliflozin (10 μM; EMPA) had no significant effect on *MT3* expression, indicating that empagliflozin does not inhibit the mitochondrial complex, as described in a previous study [10]. Statistical analysis was performed via analysis of variance (ANOVA) and Bonferroni post hoc correction. The urinary zinc levels were log(e)transformed for statistical analysis because of the skewed distribution. The data represent the means ± standard deviations (SDs). ***p*<0.01 *vs.* control.

**Supplementary Fig. 3.**


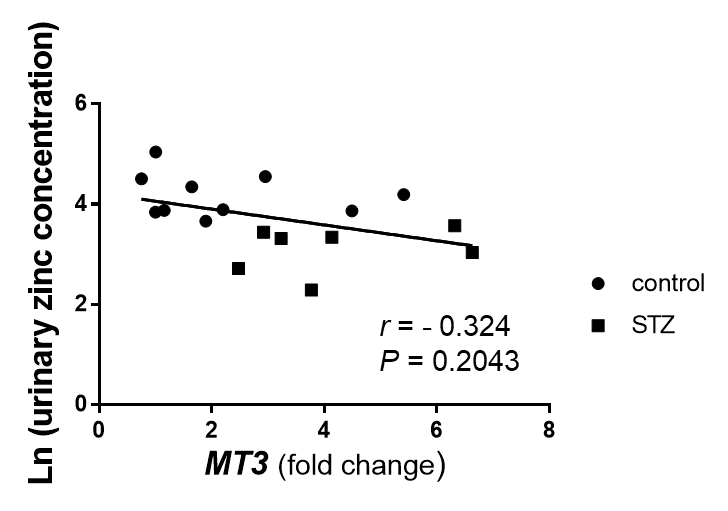

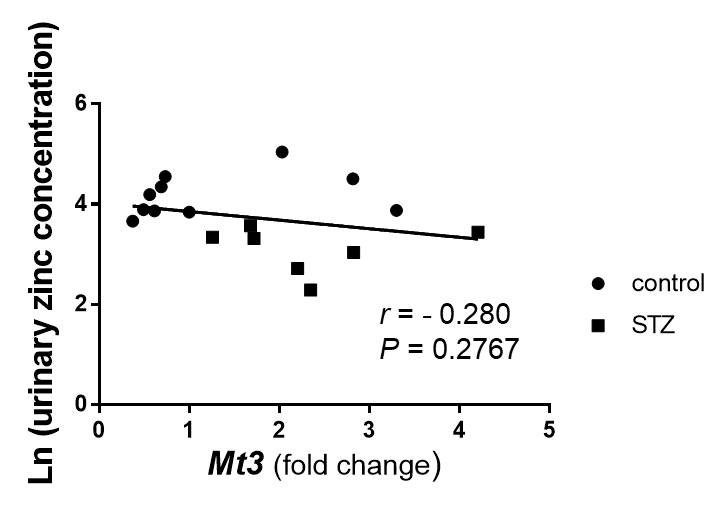


**Supplementary Fig. 3. Relationship between the urinary zinc level and *MT3* or *Mt3* expression in MT3-BACTg mice.** In contrast to the relationship between the plasma zinc level and *MT3* expression*,* as shown in Fig. 6S, no correlation was found between the urinary zinc level and *MT3* or *Mt3* expression in MT3-BACTg mice. The relationships were analyzed via simple linear regression. *P* values <0.05 were considered significant. Black circles: control MT3-BACTg (n=10). Black squares: streptozotocin-induced diabetic MT3-BACTg mice (n=7).

**Supplementary Fig. 4.**


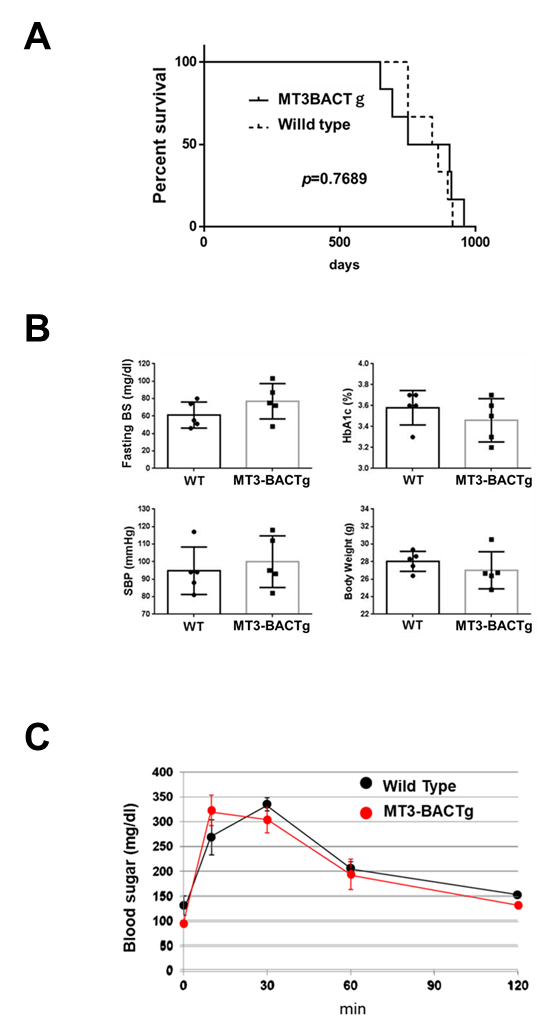


**Supplementary Fig. 4. The lifespan and glucose metabolism in aged MT3-BACTg mice. (A)** Survival status of the mice in each group, with six mice in each group. The mouse survival curves revealed no significant difference in survival between the wild-type mice and the humanized BAC transgenic mice (MT3-BACTg mice). *P* values were determined by the log-rank test. **(B)** Metabolic characteristics of two-year-old male wild-type and MT3-BACTg mice. MT3-BACTg did not result in any differences in blood glucose levels, blood pressure or body weight. **(C)** Oral glucose tolerance tests were performed on two-year-old MT3-BACTg mice. Compared with wild-type mice, MT3-BACTg mice have similar glucose levels after oral glucose administration. Fasting BG, fasting blood glucose, SBP, systolic blood pressure. The data are shown as the means ± standard deviations (SDs). The number of mice in each group was five.

**Supplementary Fig.5**


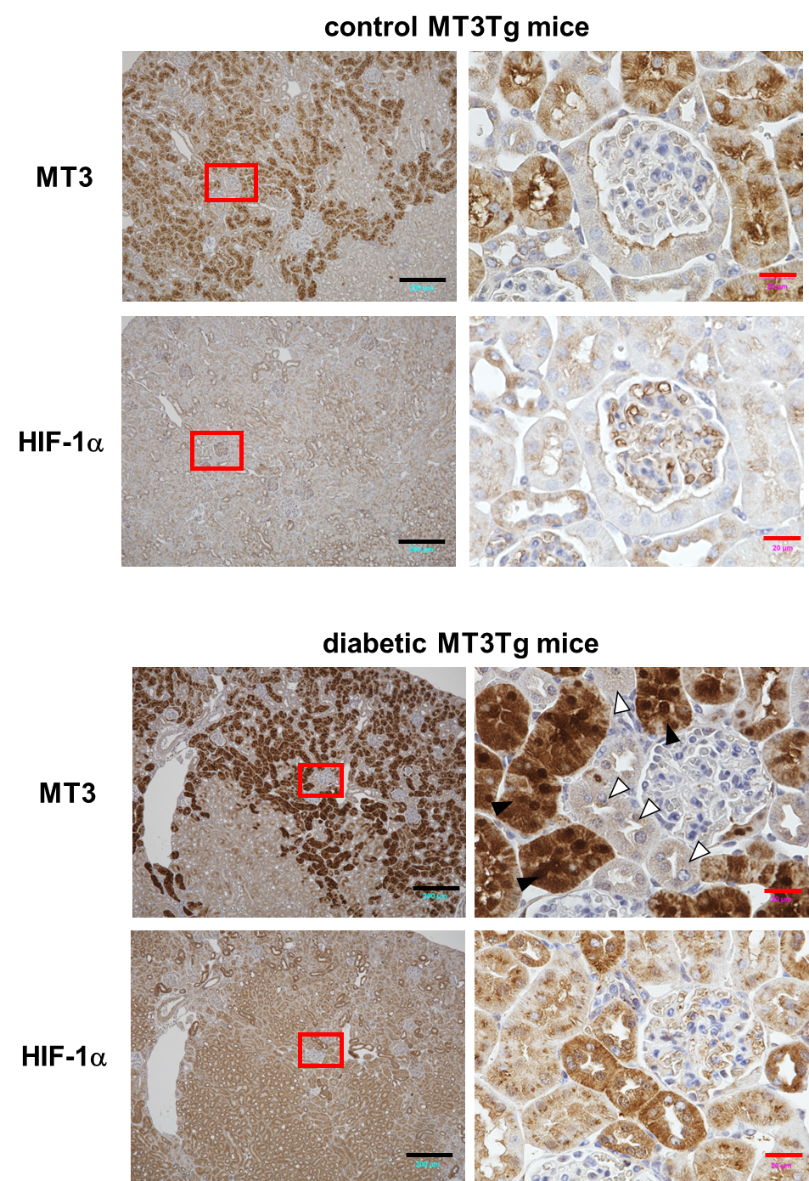


**Supplementary Fig. 5 Diabetes induced HIF-1α protein in the renal proximal tubules in proximal tubule-specific human *MT3* transgenic mice (MT3Tg), which presented no MT3 protein expression.** HIF-1α protein was only seen in no MT3-expressiong proximal tubular cells (open arrowheads), but not in MT3-expressing renal proximal cells (closed arrowheads) in diabetic MT3Tg mice. Right panels show higher magnification images of each protein staining within the red-outlined square in the left panels. Black scale bar, 200 μm; red scale bar, 20 μm.

**Supplementary Fig. 6.**

**
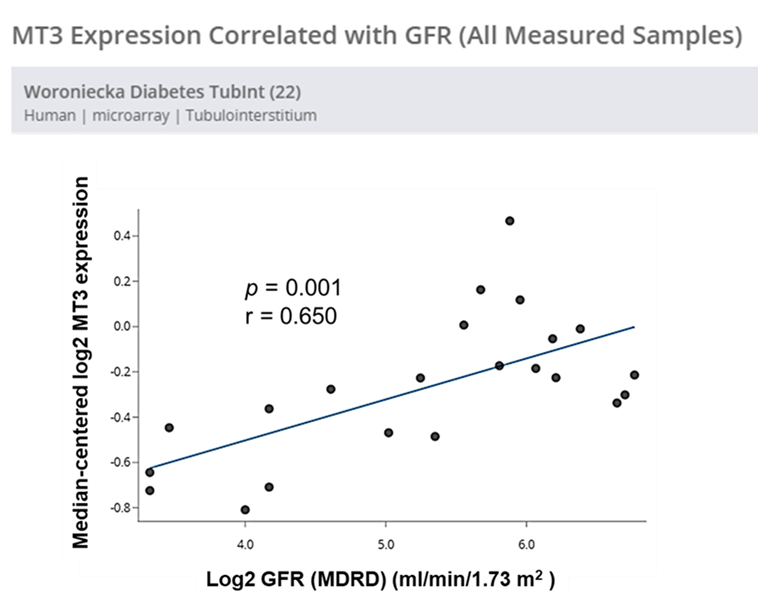
**

**Supplementary Fig. 6. Correlations between *MT3* expression and the estimated glomerular filtration rate (eGFR) in the kidney tubulointerstitium.** Both *MT3* expression and the eGFR were log2 transformed. *MT3* expression was positively correlated with the eGFR in patients with diabetic kidney disease according to the Woroniecka Diabetes TubInt Dataset [11].

**References**

1. Miyauchi K, Takiyama Y, Honjyo J, Tateno M, Haneda M. Upregulated IL-18 expression in type 2 diabetic subjects with nephropathy: TGF-β1 enhanced IL-18 expression in human renal proximal tubular epithelial cells. Diabetes research and clinical practice. 2009;83(2):190-9. doi: 10.1016/j.diabres.2008.11.018.

2. Takiyama Y, Harumi T, Watanabe J, Fujita Y, Honjo J, Shimizu N, et al. Tubular injury in a rat model of type 2 diabetes is prevented by metformin: a possible role of HIF-1α expression and oxygen metabolism. Diabetes. 2011;60(3):981-92. doi: 10.2337/db10-0655.

3. Soumura M, Kume S, Isshiki K, Takeda N, Araki S, Tanaka Y, et al. Oleate and eicosapentaenoic acid attenuate palmitate-induced inflammation and apoptosis in renal proximal tubular cell. Biochemical and biophysical research communications. 2010;402(2):265-71. doi: 10.1016/j.bbrc.2010.10.012.

4. Yamahara K, Kume S, Koya D, Tanaka Y, Morita Y, Chin-Kanasaki M, et al. Obesity-mediated autophagy insufficiency exacerbates proteinuria-induced tubulointerstitial lesions. Journal of the American Society of Nephrology : JASN. 2013;24(11):1769-81. doi: 10.1681/ASN.2012111080.

5. Pillon NJ, Sardón Puig L, Altıntaş A, Kamble PG, Casaní-Galdón S, Gabriel BM, et al. Palmitate impairs circadian transcriptomics in muscle cells through histone modification of enhancers. Life science alliance. 2023;6(1). doi: 10.26508/lsa.202201598.

6. Takeda Y, Fujita Y, Honjo J, Yanagimachi T, Sakagami H, Takiyama Y, et al. Reduction of both beta cell death and alpha cell proliferation by dipeptidyl peptidase-4 inhibition in a streptozotocin-induced model of diabetes in mice. Diabetologia. 2012;55(2):404-12. doi: 10.1007/s00125-011-2365-4.

7. Takiyama Y, Miyokawa N, Sugawara A, Kato S, Ito K, Sato K, et al. Decreased expression of retinoid X receptor isoforms in human thyroid carcinomas. J Clin Endocrinol Metab. 2004;89(11):5851-61. doi: 10.1210/jc.2003-032036.

8. Takiyama T, Sera T, Nakamura M, Hoshino M, Uesugi K, Horike SI, et al. A maternal high-fat diet induces fetal origins of NASH-HCC in mice. Sci Rep. 2022;12(1):13136. doi: 10.1038/s41598-022-17501-8.

9. Takaya K, Koya D, Isono M, Sugimoto T, Sugaya T, Kashiwagi A, et al. Involvement of ERK pathway in albumin-induced MCP-1 expression in mouse proximal tubular cells. Am J Physiol Renal Physiol. 2003;284(5):F1037-45. doi: 10.1152/ajprenal.00230.2002.

10. Albalawy WN, Youm EB, Shipman KE, Trull KJ, Baty CJ, Long KR, et al. SGLT2-independent effects of canagliflozin on NHE3 and mitochondrial complex I activity inhibit proximal tubule fluid transport and albumin uptake. Am J Physiol Renal Physiol. 2024;326(6):F1041-f53. doi: 10.1152/ajprenal.00005.2024.

11. Woroniecka KI, Park AS, Mohtat D, Thomas DB, Pullman JM, Susztak K. Transcriptome analysis of human diabetic kidney disease. Diabetes. 2011;60(9):2354-69. doi: 10.2337/db10-1181.
